# Solution-Processed Phosphorescent Organic Light-Emitting Diodes with Ultralow Driving Voltage and Very High Power Efficiency

**DOI:** 10.1038/srep12487

**Published:** 2015-07-24

**Authors:** Shumeng Wang, Xingdong Wang, Bing Yao, Baohua Zhang, Junqiao Ding, Zhiyuan Xie, Lixiang Wang

**Affiliations:** 1 State Key Laboratory of Polymer Physics and Chemistry, Changchun Institute of Applied Chemistry, Chinese Academy of Sciences, Changchun 130022, P. R. China; 2 University of Chinese Academy of Sciences, Beijing 100049, P. R. China

## Abstract

To realize power efficient solution-processed phosphorescent organic light-emitting diodes (s-PhOLEDs), the corresponding high driving voltage issue should be well solved. To solve it, efforts have been devoted to the exploitation of novel host or interfacial materials. However, the issues of charge trapping of phosphor and/or charge injection barrier are still serious, largely restraining the power efficiency (PE) levels. Herein, with the utilization of an exciplex-forming couple 4, 4′, 4″ -tris[3-methylphenyl(phenyl)amino]triphenylamine (m-MTDATA) and 1,3,5-tri(m-pyrid-3-yl-phenyl)benzene (TmPyPB), the efficient charge injection and transporting, barrier-free hole-electron recombination for the formation of the interfacial exciplex, and elimination of charge traps of phosphors in the emissive layer are realized simultaneously, resulting in a turn-on voltage of 2.36 V, a record high PE of 97.2 lm W^−1^, as well as extremely low driving voltage of 2.60 V at 100 cd m^−2^, 3.03 V at 1000 cd m^−2^ and 4.08 V at 10000 cd m^−2^. This report is the first time that the PE performance of s-PhOLED approaches 100 lm W^−1^ high level, even superior to the corresponding state-of-the-art performance of the same color vacuum-deposited PhOLED (v-PhOLED) counterpart. We anticipate this report opens a new avenue for achieving power efficient monochromatic and white s-PhOLEDs with simple structures.

Phosphorescent organic light-emitting diodes (PhOLEDs)^1^ have attracted much attention since they can harvest both singlet and triplet excitons to realize a nearly 100% internal quantum efficiency. With the exploitation of advanced multilayered device structures^2^,^3^,^4^,^5^, novel electroluminescent (EL) mechanisms^6^,^7^,^8^,^9^,^10^, and out-coupling techniques^11^,^12^, tremendous progress has been made for vacuum-deposited PhOLEDs (v-PhOLEDs) based on small molecular metallophosphors, whose power efficiency (PE) has already surpassed 100 lm W^−1^ for both monochromatic and white devices^11^,^12^,^13^,^14^. Compared with v-PhOLEDs, solution-processed PhOLEDs (s-PhOLEDs) seem to be more attractive for both academic research and industry application because they have many unique advantages^15^,^16^,^17^,^18^,^19^,^20^,^21^,^22^,^23^,^24^, such as simple device architectures, convenient high-resolution patterning over large-area substrates, and low-cost manufacturing via ink-jet printing or roll-to-roll coating. However, the performance of s-PhOLEDs^25^,^26^,^27^,^28^,^29^,^30^, especially the PE, is much lower than that of v-PhOLEDs, limiting their practical applications in energy-saving displays and lightings.

The key problem responsible for the poor PE in s-PhOLEDs is their high driving voltages, which may result from the large charge injection barrier^31^, low charge mobility^32^,^33^, heterojuction barrier necessary for the generation of bulk exciton^34^,^35^ and intense charge-trapping behavior of a phosphor in the emissive layer (EML)^36^,^37^ etc. To address these limitations, many strategies have been developed in v-PhOLEDs, such as the p-i-n structure^38^, cascade charge injection/transport multilayers^2^, and exciplex-forming co-host^14^. Unfortunately, owing to the intrinsic inter-mixing hurdle during layer-by-layer solution deposition as well as other underlying reasons including phase segregation^39^,^40^, it remains a big challenge to extend them from v-PhOLEDs to s-PhOLEDs. For example, with a mixed host composed of poly(N-vinylcarbazole) (PVK) and 1,3-bis[(4-tertbutylphenyl)-1,3,4-oxadiazolyl]phenylene (OXD-7), highly efficient yellow-emitting s-PhOLEDs were reported to give a promising current efficiency of 41.7 cd A^−1^ 41. But the power efficiency was only 12.5 lm W^−1^ because of the high driving voltage, far inferior to the co-host based v-PhOLEDs (turn-on voltage: 2.4 V; PE: 62.1 lm W^−1^)^42^. Therefore, much effort should be paid for the de nova design of the device structure and its corresponding mechanism in order to lower the driving voltage and improve the PE of s-PhOLEDs.

In this paper, we develop a solution-processed yellow-emitting electrophosphorescent device with a record high PE of 97.2 lm W^−1^ (25.2%, 74.3 cd A^−1^) by the combination of 4, 4′, 4″ -tris[3-methylphenyl(phenyl)amino]triphenylamine (m-MTDATA) and 1,3,5-tri(m-pyrid-3-yl-phenyl)benzene (TmPyPB) as an exciplex-forming couple. As shown in Fig. 1, the selection of m-MTDATA with high-lying highest occupied molecular orbital (HOMO) level as the hole-transporting host and TmPyPB with good electron mobility as the electron-transporting layer (ETL) can be able to realize the effective injection and transporting for both holes and electrons, avoiding the high driving voltage issue caused by the large charge injection barrier and low charge mobility. In addition, carriers are not required to surmount the heterojuction barrier, but directly recombine at the m-MTDATA/TmPyPB interface to form the localized excited-state exciplex, which is a barrier-free process. Most importantly, the energy of the formed exciplex is delivered to the dopant mainly by energy transfer, and the unwanted charge trapping effect is successfully eliminated. Consequently, an extremely low turn-on voltage of 2.36 V is achieved, and at a luminance of 100, 1000 and 10000 cd m^−2^, the driving voltage is found to be 2.60, 3.03 and 4.08 V, respectively. To the best of our knowledge, these values are among the lowest ever reported for s-PhOLEDs, and the maximum PE is comparable to the current state-of-the art of v-PhOLEDs for the first time. The versatility of this strategy is further demonstrated by the achievement of ultralow voltage driving green- and red-emitting s-PhOLEDs, revealing a peak PE of 81.1 lm W^−1^ (18.1%, 62.0 cd A^−1^) and 29.0 lm W^−1^ (16.3%, 22.2 cd A^−1^), respectively. Systematic research about the device operating mechanism will be performed subsequently, which can give us a clear picture of our concept.

## Results and Discussion

Single carrier devices of pure m-MTDATA and TmPyPB films were firstly explored, and their current density-voltage (J-V) characteristics are shown in Figure S2. For the hole-only device of m-MTDATA, the hole current initially starts at the voltage onset, and then rises rapidly with the increasing voltage, indicative of the effective hole injection and transporting. According to the literature^43^, m-MTDATA could form ohmic contact with the anode to facilitate the hole injection. Meanwhile, its higher HOMO level (−5.10 eV, Table S2) relative to dopant is beneficial to remove the negative hole trapping effect and favor the hole transporting in the EML. On the other hand, the cathode structure of TmPyPB/LiF/Al is also efficient for electron injection and transporting, which is verified by the sharply growth of the electron current with a low threshold voltage of 0.4 V (See electron-only device of TmPyPB in Figure S2b). In fact, the nitrogen atom in TmPyPB could coordinate with metal to guarantee efficient electron injection from LiF/Al, and the electron mobility of TmPyPB is as high as 1 × 10^−3^ cm^2^ V^−1^ s^−1^ 32, ensuring the efficient electron transporting in the ETL.

**Figure 2:**
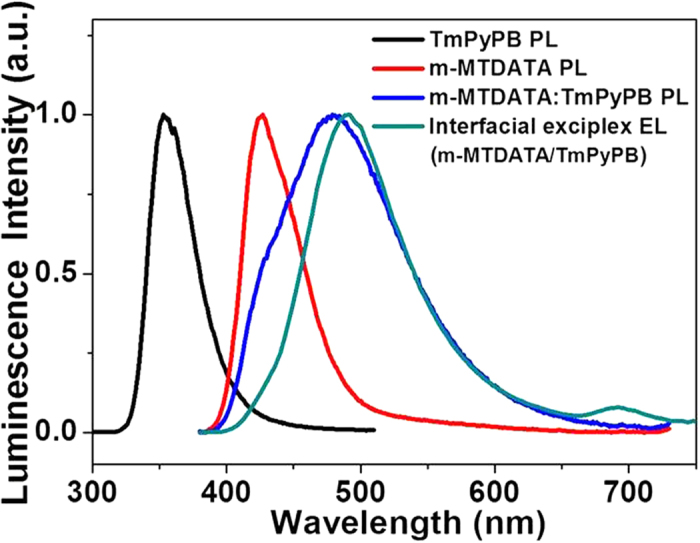
Photophysical characteristics of different samples. Normalized PL spectra of m-MTDATA, TmPyPB, and m-MTDATA:TmPyPB (1:1, mol/mol) blended films, together with normalized EL spectrum of m-MTDATA/TmPyPB bilayer device.

Then we identify the exciplex nature between m-MTDATA and TmPyPB prior to demonstrate the exciton formation process in the EL device. The photoluminescent (PL) spectra of m-MTDATA, TmPyPB and m-MTDATA:TmPyPB (1:1, mol/mol) blended films together with the EL spectrum of the m-MTDATA/TmPyPB bilayer device are shown in Fig. 2. In comparison to the individual PL spectra of m-MTDATA and TmPyPB films, the m-MTDATA:TmPyPB blended film exhibits a new red-shifted broad emission with a peak at 490 nm, accompanied by a PL shoulder from m-MTDATA. The energy of the new emission (2.54 eV) is close to the energy gap between the HOMO level of m-MTDATA and the lowest unoccupied molecular orbital (LUMO) level of TmPyPB, indicating that m-MTDATA and TmPyPB could form exciplex excited state^44^,^45^,^46^,^47^. And the existence of the partial m-MTDATA emission is reasonable when considering that the bulk m-MTDATA excitons are firstly formed under optical excitation. The possible phase separation in the m-MTDATA and TmPyPB blended film (with large domain-size) via solution-process may lead to inefficient exciton diffusion and incomplete energy transfer from the m-MTDATA excited state to the localized exciplex formed at the m-MTDATA:TmPyPB heterojunction interface^48^,^49^,^50^. By contrast, the m-MTDATA emission is almost absent in the EL spectrum of the m-MTDATA/TmPyPB bilayer device. This is because the exciplex is directly produced from the opposite charge separated states under electric excitation^34^,^35^. Moreover, the EL exciplex emission is slightly red-shifted compared with m-MTDATA:TmPyPB (1:1, mol/mol), which may be caused by the PL contribution from m-MTDATA. As shown in Figure S3, with the decreasing ratio of m-MTDATA to TmPyPB, the m-MTDATA emission is reduced, and the emissive maximum of the m-MTDATA:TmPyPB mixture gradually moves to match the EL exciplex. An additional shoulder peaked at 694 nm that is attributable to the m-MTDATA electromer is also found, and disappears after doping with phosphorescent dyes (Figure S4 and Figure S16).

Based on the above-mentioned research, we can safely deduce that upon the accumulation of the opposite charges at the m-MTDATA/TmPyPB heterojunction, they subsequently recombine via a barrier-free hole-electron capture to generate the exciplex across the interface. This process III can avoid the band-edge offset induced by the formation of the bulk m-MTDATA excitons, and energetically lower the device driving voltages. Although a transition from the exciplex to bulk m-MTDATA excitons does really occur at a certain high electric field (e.g., at 13 V, Figure S5), we note that the exciplex generation is the sole way at a practical working voltage less than 5 V (corresponding to a luminance of ca. 20000 cd m^−2^).

**Figure 3:**
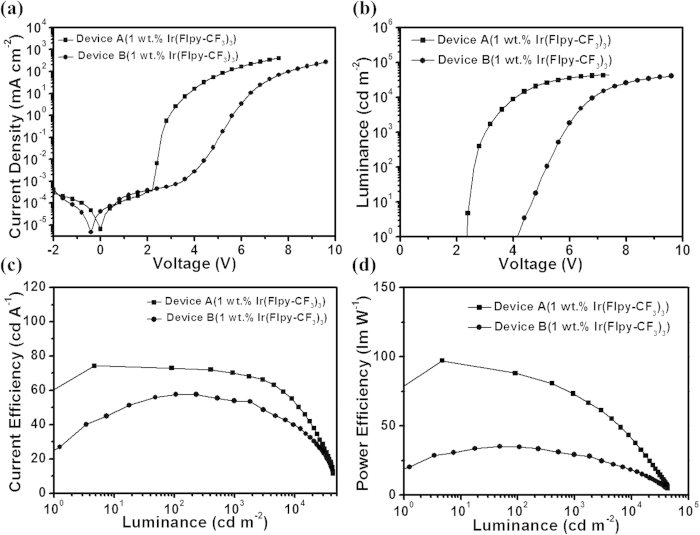
Performance characteristics of solution-processed OLEDs. Current density-voltage (**a**), Luminance-voltage (**b**), Current efficiency-luminance (**c**) and Power efficiency-luminance (d) characteristics for device A (1 wt.% Ir(Flpy-CF_3_)_3_) and B (1 wt.% Ir(Flpy-CF_3_)_3_).

When phosphors are dispersed into the m-MTDATA to constitute the EML, the exciplex emission could be quenched completely (Figure S16) due to the efficient energy transfer from the exciplex to dopant. During this process IV, it should be noted that, the application of the interfacial exciplex of m-MTDATA/TmPyPB could eliminate the unwanted charge-trapping behavior of a narrow-bandgap dye in s-PhOLEDs, decrease the driving voltage as much as possible, and thus greatly improve the device performance. For example, with an iridium complex containing 5-trifluoromethyl-2-(9,9-diethylfluoren-2-yl)pyridine ligand (Ir(Flpy-CF_3_)_3_) as the dopant, we simultaneously prepare two types of yellow-emitting s-PhOLEDs for comparison: ITO/PEDOT:PSS(40 nm)/m-MTDATA:Ir(Flpy-CF_3_)_3_(40 nm)/TmPyPB(55 nm)/LiF(0.5 nm)/Al(100 nm) (denoted as device A), and ITO/PEDOT:PSS(40 nm)/m-MTDATA:TmPyPB (1:1, mol:mol): Ir(Flpy-CF_3_)_3_ (40 nm)/TmPyPB(55 nm)/LiF(0.5 nm)/Al(100 nm) (denoted as device B). All the device parameters are the same for device A and B except for the EML composition. As for device A, a pure m-MTDATA is used as the host in the EML, and the exciplex is formed at the interface between m-MTDATA and TmPyPB as discussed above. While for device B, a binary m-MTDATA:TmPyPB host system is utilized in the EML, and the generation region of the exciplex is extended to the whole EML (Figure S6 and S7). Fig. 3 presents the luminance-voltage and power efficiency-luminance curves of these two devices. As can be clearly seen, device A displays a much lower turn-on voltage of 2.36 V than that of device B (4.15 V). Correspondingly, a state-of-art PE of 97.2 lm W^−1^ (25.2%, 74.3 cd A^−1^) is obtained for device A, which is comparable to or even higher than most high-performance v-PhOLEDs (Table S1)^42^,^51^,^52^. Moreover, compared with device B (35.2 lm W^−1^), the PE is improved by about three folds, which may be ascribed to the extremely low driving voltage as a result of the insignificant charge trapping effect in device A.

**Figure 4:**
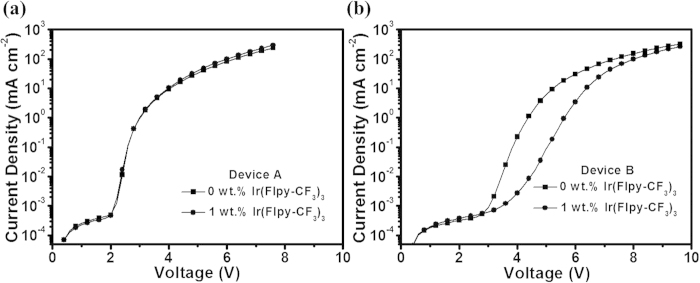
Current density-voltage characteristics of different devices. device A (0 wt.% and 1 wt.% Ir(Flpy-CF_3_)_3_) (**a**), and device B (0 wt.% and 1 wt.% Ir(Flpy-CF_3_)_3_) (**b**).

The voltage dependence of the current density for devices with and without dopant is then investigated to demonstrate whether the charge trap effect does exist. As depicted in Fig. 4a, device A in the absence of the dopant shows an abrupt current turn-on at a very low voltage of 2.1 V, and then the current density sharply increases with the increasing of voltage. It is reasonable since both holes and electrons are capable of efficient injecting and transporting through the pure m-MTDATA and TmPyPB to reach at their interface and form the recombination current. Additionally, the J-V curve remains nearly identical after the addition of Ir(Flpy-CF_3_)_3_, indicative of the ignorable charge traps in this device configuration. As discussed above, the barrier-free hole-electron capture is accomplished at the m-MTDATA/TmPyPB interface to generate the exciplex excitons. Accordingly, the injected electrons can not penetrate from TmPyPB into the bulk EML, excluding the possible bulk electron trapping of Ir(Flpy-CF_3_)_3_ in the EML during device operation. It well explains the charge trapping-free behavior of device A with Ir(Flpy-CF_3_)_3_. In contrast, a quite different behavior is observed for the device B structure. For example, device B without dopant exhibits a gentle current turn-on at a higher voltage of 2.8 V, and then the current density slowly increases as the voltage grows (Fig. 4b). The reason lies in that the carrier transport may become more difficult in the blended co-host than in the individual host, which correlates well with the decreased hole and electron current of m-MTDATA:TmPyPB blended co-host layer relative to pure m-MTDATA and TmPyPB layers (Figure S8). Upon doping only 1 wt.% Ir(Flpy-CF_3_)_3_ into the EML, the current density is found to be reduced obviously in a voltage range of 3–8 V. This means the existence of distinct charge-trapping of Ir(Flpy-CF_3_)_3_ in the corresponding device. Unlike device A, whose recombination region is at the m-MTDATA/TmPyPB interface, the exciton formation region of device B is dominated in the bulk EML comprising of the m-MTDATA:TmPyPB blended host matrix and the Ir(Flpy-CF_3_)_3_ dopant. In this case, when electrons are transported into the EML, strong electron trapping does appear owing to the deep electron trap-depth of ca. 0.6 eV for Ir(Flpy-CF_3_)_3_ with respect to TmPyPB. The observed difference between device A and B well portrays the superiority of the interfacial exciplex because it can restrain the negative effect from the charge traps, and thereby lower the device driving voltage.

Compared with the control device B, device A is also advantageous to avoid the exciton quenching from the PEDOT:PSS layer^53^,^54^ since excitons are formed at the m-MTDATA/TmPyPB interface. Secondly, the exciton generation possibility is believed to be efficient due to the effective injection and transporting for both holes and electrons. Thirdly, the triplet energies of m-MTDATA (2.66 eV), TmPyPB (2.80 eV) and m-MTDATA:TmPyPB exciplex (2.49 eV) (Figure S9 and S10) are higher than that of Ir(Flpy-CF_3_)_3_ (2.24 eV), ensuring that the generated excitons can be efficiently harvested by Ir(Flpy-CF_3_)_3_ and the triplet exciton quenching by Ir(Flpy-CF_3_)_3_ is minimized. All these factors contribute to the achieved promising external quantum efficiency (EQE) of 25.2% for device A (Table 1 and Figure S11). Furthermore, the EQE displays a very slow roll-off, e.g., remaining at 24.8, 23.7 and 18.1% corresponding to a luminance of 100, 1000 and 10000 cd m^−2^, respectively. As determined by Greenham *et al.*^55^, the carrier density for the exciplex formation at the heterojunction interface is actually low, and the possible polaron-exciton quenching may be prevented, resulting in a gentle decay of the device efficiency.

In addition, the EL spectrum of device A is mainly from Ir(Flpy-CF_3_)_3_ and independent of the driving voltage (Figure S16a), implying that the proposed exciplex-to-dopant resonant energy transfer is very efficient even at high luminance. To illustrate this point, four other devices with a similar configuration to device A are fabricated (Figure S12a), where the Ir(Flpy-CF_3_)_3_-doped m-MTDATA EML is separated from the TmPyPB ETL by a neat m-MTADA layer (1, 3, 5 or 10 nm). With the increasing thickness of the non-doped m-MTADA layer, the intensity of exciplex emission is found to be enhanced (Figure S12b), and correspondingly the device performance is decreased (Figure S13 and Table S3). Nevertheless, we note that, when the thickness of the non-doped m-MTADA layer is up to 5 nm, the EQE at a 100 cd m^−2^ luminance for these devices still remains at above 20%, and the Ir(Flpy-CF_3_)_3_ emission dominates the whole EL spectrum. This indicates that the exciton energy on the interfacial exciplex could be effectively harvested by the dopant.

At last, we expand our concept to other emission colors. Considering the higher triplet level of the m-MTDATA:TmPyPB exciplex relative to green and red phosphors, in principle, the ultralow voltage driving green- and red-emitting s-PhOLEDs are within our expectation. Therefore, device C and D are fabricated by doping the home-made green G0^56^ and red Ir(TPAPQ)_2_acac^57^ into m-MTDATA, respectively (Figure S14). Similar to the yellow-emitting device A, they both possess ultralow operational voltage and high PE. At a luminescence of 1000 cd m^−2^, for instance, the voltage and PE are 3.03 V and 62.5 lm W^−1^ for device C, and 3.42 V and 18.8 lm W^−1^ for device D (Table 1). The comprehensive performance is the highest for green- and red-emitting s-PhOLEDs reported so far^18^,^19^,^21^,^22^,^23^,^24^,^25^,^26^,^27^,^28^,^29^,^30^. Interestingly, both of device C and D exhibit the same J-V characteristics and turn-on voltages as that of device A, independent of the phosphors used (Figure S15). These observations further confirm that the energy transfer from the interfacial exciplex other than the charge trapping is the dominant EL contribution.

## Conclusions

In conclusion, we have successfully developed a power-efficient yellow-emitting s-PhOLEDs with ultralow driving voltage for the first time. With the utilization of an exciplex-forming couple m-MTDATA and TmPyPB, the efficient charge injection and transporting, barrier-free hole-electron recombination for the formation of the interfacial exciplex, and elimination of charge traps in the EML can be realized simultaneously, resulting in a turn-on voltage of 2.36 V, a record high PE of 97.2 lm W^−1^, as well as extremely low driving voltage of 2.60 V at 100 cd m^−2^, 3.03 V at 1000 cd m^−2^ and 4.08 V at 10000 cd m^−2^. Meanwhile, ultralow voltage driving green- and red-emitting s-PhOLEDs are also demonstrated based on the same concept, showing a maximum PE of 81.1 and 29.0 lm W^−1^, respectively. The remarkable progress has further promoted us to expand this design strategy to solution-processed blue and white devices, and the related work is under way.

## Methods

### Material information

Poly(ethylenedioxythiophene):poly(styrenesulfonate) (PEDOT:PSS) (Clevios P AI4083), m-MTDATA and TmPyPB were purchased from Heraeus Precious Metals GmbH Co. KG and Luminescence Technology Corp., respectively. The phosphors Ir(Flpy-CF_3_)_3_^58^, G0^56^ and Ir(TPAPQ)_2_acac^57^ were synthesized in our laboratory according to the literature methods.

### Cyclic voltammetry (CV) measurements

CV measurements were carried out on an EG & G 283 (Princeton Applied Research) potentiostat/galvanostat system. Solvents of CV measurements were chosen according to solubility and electrochemical window of different materials, that is *E*_ox_ of m-MTDATA, G0, Ir(Flpy-CF_3_)_3_, and Ir(TPAPQ)_2_acac in dichloromethane, *E*_ox_ of TmPyPB and *E*_red_ of TmPyPB, G0, Ir(Flpy-CF_3_)_3_, and Ir(TPAPQ)_2_acac in acetonitrile. All samples were tested at a scanning rate of 100 mV s^−1^. The supporting electrolyte was 0.1 M tetrabutylammonium perchorate (n-Bu_4_NClO_4_). Regarding to energy levels of ferrocene/ferrocenium couple reference (4.8 eV relative to the vacuum level) the HOMO and LUMO energy levels were calculated according to the following two equations: *E*_HOMO_ = −e(4.8 V + *E*_ox_), and *E*_LUMO_ = −e(4.8 V + *E*_red_), except for LUMO energy level of m-MTDATA which was calculated according to the equation: *E*_LUMO_ = *E*_HOMO_ + *E*_g_. Here, *E*_ox_ and *E*_red_ were gotten from the onset of the oxidation and reduction potential, and *E*_g_ was the optical band gap estimated from the onset of the absorption spectra. Furthermore, the HOMO and the lowest unoccupied molecular orbital (LUMO) levels of all used functional materials were calibrated with m-MTDATA as the reference^59^, whose HOMO level is −5.10 eV measured by ultraviolet photoemission spectroscopy (UPS).

### Photophysical and atomic force microscopy (AFM) mesasurements

UV-Vis absorption spectra were measured using Perkin-Elmer Lambda 35 Uv-Vis spectrometer. Film photoluminescent (PL) spectra were measured using Edinburgh FLS920 PL spectrometer. Solution PL spectra were measured using Perkin-Elmer LS 50B PL spectrometer. Solution PL quantum efficiency was measured in argon atmosphere by a relative method using fac-Ir(ppy)_3_ (Φ_p_ = 0.40 in toluene) as the standard. AFM measurements were carried out using Veeco Instruments in the tapping mode with a 2 N m^−1^ probe in the atmospheric environment. For the sample preparation, the pre-cleaned indium tin oxide (ITO) substrate was ultra violet-ozone (UVO) treated for 25 min. A 40 nm thick PEDOT:PSS was spin-coated from an aqueous dispersion of PEDOT:PSS (Clevios P AI4083) at a spin speed of 5000 rpm and was annealed at 120 °C for 30 min. in air condition. Finally, the m-MTDATA:Dopant layer was spin-coated from its fresh chlorobenzene solution (10 mg ml^−1^) at a spin speed of 1800 rpm and was annealed at 100 °C for 30 min. at N_2_ atmosphere in a glove box.

### Device preparation and characterization

The s-PhOLEDs have the configuration of ITO/PEDOT:PSS(40 nm)/emissive layer (EML)(40 nm)/TmPyPB(55 nm)/LiF(0.5 nm)/Al(100 nm), in which the EML consists of m-MTDATA:Ir(Flpy-CF_3_)_3_(1 wt.%) for device A, m-MTDATA:TmPyPB(1:1, mol/mol):Ir(FIpy-CF_3_)_3_(1 wt.%) for device B, m-MTDATA:G0(10 wt.%) for device C, and m-MTDATA:Ir(TPAPQ)_2_acac(5 wt.%) for device D. For the device fabrication, a 40 nm-thick PEDOT:PSS layer was spin-coated from an aqueous dipersion of PEDOT:PSS (Clevios P AI4083) onto the pre-cleaned and UVO treated ITO substrate at a spin speed of 5000 rpm, and then annealed at 120 °C for 30 min. in air condition. Subsequently, the EML was spin-coated from its fresh chlorobenzene solution(10 mg ml^−1^) at a spin speed of 1800 rpm and then annealed at 100 °C for 30 min. to remove the residual solvent at N_2_ atmosphere in a glove box. Finally, the structure of TmPyPB(55 nm)/LiF(0.5 nm)/Al(100 nm) was thermally deposited in sequence in a vacuum chamber at a pressure less than 4 × 10^−4^ Pa through a shadow mask with an array of 14 mm^2^ openings. The current density-voltage-luminance (J–V–L) characteristics were measured using a Keithley source measurement unit (Keithley 2400 and Keithley 2000) with a calibrated silicon photodiode. The EL spectra of the devices were measured using a SpectraScan PR650 spectrophotometer. All measurements were carried out at room temperature under ambient conditions. External quantum efficiency (EQE) of the devices were calculated from the luminance, current density and EL spectra, assuming a Lambertian distribution. Low temperature EL spectra were measured with an Oxford Instrument Optistat DN-V cryostat system coupled with a PR650 spectrophotometer. Temperatures were controlled within ± 0.1 K by an Oxford Instrument ITC503S temperature controller.

### Hole-only and electron-only devices

The structure for hole-only devices is ITO/PEDOT:PSS(40 nm)/m-MTDATA or m-MTDATA:TmPyPB(1:1, mol/mol)(70 nm)/Au(50 nm). For the fabrication of the hole-only device, a 40 nm-thick PEDOT:PSS (Clevios P AI4083) layer was spin-coated onto the pre-cleaned ITO substrate and then annealed at 120 °C for 30 min in air condition. Subsequently, m-MTDATA or m-MTDATA:TmPyPB(1:1, mol/mol)(70 nm) was spin-coated from its fresh chlorobenzene solution and then annealed at 100 °C for 30 min to remove residual solvent at N_2_ atmosphere. Finally, metal Au cathode was thermally deposited in a vacuum chamber at a pressure less than 4 × 10^−4^ Pa through a shadow mask with an array of 14 mm^2^ openings. The device structure for electron-only device is Glass/PEDOT:PSS(40 nm)/Al(50 nm)/TmPyPB or m-MTDATA:TmPyPB(1:1, mol/mol)(40 nm)/TmPyPB(55 nm)/LiF(0.5 nm)/Al(l00 nm)^60^. Firslty, a 40 nm-thick PEDOT:PSS (Clevios P AI4083) layer was spin-coated onto the pre-cleaned glass substrate and then annealed at 120 °C for 30 min in air condition. And then, a 50 nm thick Al was thermally deposited in a vacuum chamber at a base pressure less than 4 × 10^−4^ Pa. Subsequently, for the m-MTDATA:TmPyPB(1:1, mol/mol) device, m-MTDATA:TmPyPB(1:1, mol/mol) (40 nm) was spin-coated from its fresh chlorobenzene solution and then annealed at 100 °C for 30 min to remove residual solvent at N_2_ atmosphere, and for the TmPyPB device, a 40 nm thick TmPyPB was thermally deposited in a vacuum chamber at a base pressure less than 4 × 10^−4^ Pa. The structure of TmPyPB(55 nm)/LiF(0.5 nm)/Al(l00 nm) was thermally deposited in sequence to accomplish the device preparation, at a base pressure less than 4 × 10^−4^ Pa through a shadow mask with an array of 14 mm^2^ openings.

## Additional Information

**How to cite this article**: Wang, S. *et al.* Solution-Processed Phosphorescent Organic Light-Emitting Diodes with Ultralow Driving Voltage and Very High Power Efficiency. *Sci. Rep.* **5**, 12487; doi: 10.1038/srep12487 (2015).

## Supplementary Material

Supplementary Information

## Figures and Tables

**Figure 1:**
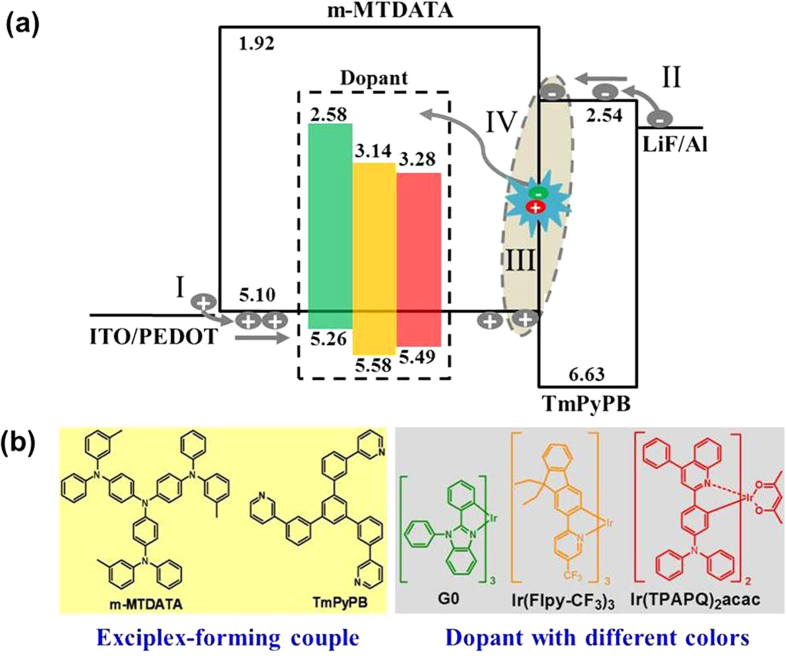
Device operating mechanism and molecular structures. (**a**) The proposed device operating mechanism including I: effective hole injection and transporting; II: effective electron injection and transporting; III: barrier-free hole-electron recombination for the formation of exciplex; and IV: energy transfer from interfacial exciplex to phosphor; (**b**) Molecular structures of functional materials used.

**Table 1:** Summary of device performances.

Device	Voltage (V)	LE (cd A^−1^)	PE (lm W^−1^)	EQE (%)		CIE (x, y)
	1/100/1000/10000 cd m^−2^	Max/100/1000/10000 cd m^−2^	Max/100/1000/10000 cd m^−2^	Max/100/1000/10000 cd m^−2^	Max Luminance (cd m^−2^)	
A (yellow)	2.36/2.60/3.03/4.08	74.3/72.6/70.1/53.5	97.2/87.7/72.5/40.8	25.2/24.8/23.7/18.1	43085	(0.52, 0.47)
B (yellow)	4.15/5.18/5.80/6.82	57.6/57.4/53.9/39.7	35.2/34.8/29.1/18.0	19.5/19.4/18.3/13.4	42476	(0.52, 0.47)
C (green)	2.36/2.60/3.03/4.10	62.0/61.6/60.7/49.7	81.1/73.4/62.5/37.8	18.1/18.1/17.8/14.6	41539	(0.35, 0.59)
D (red)	2.36/2.75/3.42/5.50	22.2/21.3/20.4/10.7	29.0/24.4/18.8/5.9	16.3/15.9/14.9/8.0	17902	(0.64, 0.36)

## References

[b1] Reineke S., Thomschke M., Luessem B. & Leo K. White organic light-emitting diodes: status and perspective. Rev. Mod. Phys. 85, 1245–1293 (2013).

[b2] Su S. J., Gonmori E., Sasabe H. & Kido J. Highly efficient organic blue- and white-light-emitting devices having a carrier- and exciton-confining structure for reduced efficiency roll-off. Adv. Mater. 20, 4189–4194 (2008).

[b3] Wang Q. *et al.* A non-doped phosphorescent organic light-emitting device with above 31% external quantum efficiency. Adv. Mater. 26, 8107–8113 (2014). 25219957 10.1002/adma.201402947

[b4] Sasabe H. *et al.* Extremely low operating voltage green phosphorescent organic light-emitting devices. Adv. Funct. Mater. 23, 5550–5555 (2013).

[b5] Lee J. H. *et al.* An exciplex forming host for highly efficient blue organic light emitting diodes with low driving voltage. Adv. Funct. Mater. 25, 361–366 (2015).

[b6] Uoyama H., Goushi K., Shizu K., Nomura H. & Adachi C. Highly efficient organic light-emitting diodes from delayed fluorescence. Nature 492, 234–238 (2012). 23235877 10.1038/nature11687

[b7] Zhang Q. *et al.* Nearly 100% internal quantum efficiency in undoped electroluminescent devices employing pure organic emitters. Adv. Mater. 27, 2096–2100 (2015). 25678335 10.1002/adma.201405474

[b8] Zhang D. *et al.* High-efficiency fluorescent organic light-emitting devices using sensitizing hosts with a small singlet-triplet exchange energy. Adv. Mater. 26, 5050–5055 (2014). 24944186 10.1002/adma.201401476

[b9] Wang K. *et al.* High-performance red, green, and blue electroluminescent devices based on blue emitters with small singlet-triplet splitting and ambipolar transport property. Adv. Funct. Mater. 23, 2672–2680 (2013).

[b10] Liu X. K. *et al.* Nearly 100% triplet harvesting in conventional fluorescent dopant-based organic light-emitting devices through energy transfer from exciplex. Adv. Mater. 27, 2025–2030 (2015). 25676085 10.1002/adma.201500013

[b11] Reineke S. *et al.* White organic light-emitting diodes with fluorescent tube efficiency. Nature 459, 234–238 (2009). 19444212 10.1038/nature08003

[b12] Ou Q. D. *et al.* Extremely efficient white organic light-emitting diodes for general lighting. Adv. Funct. Mater. 24, 7249–7256 (2004).

[b13] Seo S. *et al.* Exciplex-triplet energy transfer: a new method to achieve extremely efficient organic light-emitting diode with external quantum efficiency over 30% and drive voltage below 3V. Jpn. J. Appl. Phys. 53, 042102 (2014).

[b14] Park Y. S. *et al.* Exciplex-forming co-host for organic light-emitting diodes with ultimate efficiency. Adv. Funct. Mater. 23, 4914–4920 (2013).

[b15] Wu H., Ying L., Yang W. & Cao Y. Progress and perspective of polymer white light-emitting devices and materials. Chem. Soc. Rev. 38, 3391–3400 (2009). 20449058 10.1039/b816352a

[b16] Xia S. *et al.* Printable phosphorescent organic light-emitting devices. J. SID 17, 167–172 (2009).

[b17] Kamtekar K. T., Monkman A. P. & Bryce M. R. Recent advances in white organic light-emitting materials and devices (WOLEDs). Adv. Mater. 22, 572–582 (2010). 20217752 10.1002/adma.200902148

[b18] Rehmann N., Hertel D., Meerholz K., Becker H. & Heun S. Highly efficient solution-processed phosphorescent multilayer organic light-emitting diodes based on small-molecule hosts. Appl. Phys. Lett. 91, 103507 (2007).

[b19] Perumal A. *et al.* High-efficiency, solution-processed, multilayer phosphorescent organic light-emitting diodes with a copper thiocyanate hole-injection/hole-transport layer. Adv. Mater. 27, 93–100 (2015). 25382072 10.1002/adma.201403914 PMC4315901

[b20] Lee C. W. & Lee J. Y. High quantum efficiency in solution and vacuum processed blue phosphorescent organic light emitting diodes using a novel benzofuropyridine-based bipolar host material. Adv. Mater. 25, 596–600 (2013). 23136111 10.1002/adma.201203180

[b21] Jou J. H. *et al.* High-efficiency wet- and dry-processed green organic light emitting diodes with a novel iridium complex-based emitter. Adv. Opt. Mater. 1, 657–667 (2013).

[b22] Cho Y. J., Yook K. S. & Lee J. Y. High efficiency in a solution-processed thermally activated delayed-fluorescence device using a delayed-fluorescence emitting material with improved solubility. Adv. Mater. 26, 6642–6646 (2014). 25179914 10.1002/adma.201402188

[b23] Cai M. *et al.* High-efficiency solution-processed small molecule electrophosphorescent organic light-emitting diodes. Adv. Mater. 23, 3590–3596 (2011). 21728190 10.1002/adma.201101154

[b24] Aizawa N., *et al.* Solution-processed multilayer small-molecule light-emitting devices with high-efficiency white-light emission. Nat. Commun. 5, 5756 (2014). 25519692 10.1038/ncomms6756

[b25] Zhou G., Wong W. Y., Yao B., Xie Z. & Wang L. Triphenylamine-dendronized pure red iridium phosphors with superior OLED efficiency/color purity trade-offs. Angew. Chem. Int. Ed. 46, 1149–1151 (2007). 10.1002/anie.200604094 17200970

[b26] Wang X. *et al.* Solution-processible 2,2‘-dimethyl-biphenyl cored carbazole dendrimers as universal hosts for efficient blue, green, and red phosphorescent OLEDs. Adv. Funct. Mater. 24, 3413–3421 (2014).

[b27] Li J., Zhang T., Liang Y. & Yang R. Solution-processible carbazole dendrimers as host materials for highly efficient phosphorescent organic light-emitting diodes. Adv. Funct. Mater. 23, 619–628 (2013).

[b28] Aizawa N., Pu Y. J., Sasabe H. & Kido J. Solution-processable carbazole-based host materials for phosphorescent organic light-emitting devices. Org. Electron. 13, 2235–2242 (2012).

[b29] Duan L., *et al.* Solution processable small molecules for organic light-emitting diodes. J. Mater. Chem. 20, 6392–6407 (2010).

[b30] Yook K. S. & Lee J. Y. Small molecule host materials for solution processed phosphorescent organic light-emitting diodes. Adv. Mater. 26, 4218–4233 (2014). 24807691 10.1002/adma.201306266

[b31] Huang F., Cheng Y. J., Zhang Y., Liu M. S. & Jen A. K. Y. Crosslinkable hole-transporting materials for solution processed polymer light-emitting diodes. J. Mater. Chem. 18, 4495–4509 (2008).

[b32] Su S. J., Chiba T., Takeda T. & Kido J. Pyridine-containing triphenylbenzene derivatives with high electron mobility for highly efficient phosphorescent OLEDs. Adv. Mater. 20, 2125–2130 (2008).

[b33] Su S. J., Sasabe H., Pu Y. J., Nakayama K. & Kido J. Tuning energy levels of electron-transport materials by nitrogen orientation for electrophosphorescent devices with an ‘Ideal’ operating voltage. Adv. Mater. 22, 3311–3316 (2010). 20552601 10.1002/adma.200904249

[b34] Morteani A. C. *et al.* Barrier-free electron-hole capture in polymer blend heterojunction light-emitting diodes. Adv. Mater. 15, 1708–1712 (2013).

[b35] Morteani A. C., Ho P. K. H., Friend R. H. & Silva C. Electric field-induced transition from heterojunction to bulk charge recombination in bilayer polymer light-emitting diodes. Appl. Phys. Lett. 86, 163501 (2005).

[b36] Tsung K. K. & So S. K. Carrier trapping and scattering in amorphous organic hole transporter. Appl. Phys. Lett. 92, 103315 (2008).

[b37] Lee J. H., Lee S., Yoo S. J., Kim K. H. & Kim J. J. Langevin and trap-assisted recombination in phosphorescent organic light emitting diodes. Adv. Funct. Mater. 24, 4681–4688 (2014).

[b38] Walzer K., Maennig B., Pfeiffer M. & Leo K. Highly efficient organic devices based on electrically doped transport layers. Chem. Rev. 107, 1233–1271 (2007). 17385929 10.1021/cr050156n

[b39] Meerholz K. Device physics—enlightening solutions. Nature 437, 327–328 (2005). 16163334 10.1038/437327a

[b40] Noh Y. Y., Lee C. L., Kim J. J. & Yase K. Energy transfer and device performance in phosphorescent dye doped polymer light emitting diodes. J. Chem. Phys. 118, 2853–2864 (2003).

[b41] Ye T., Shao S., Chen J., Wang L. & Ma D. Efficient phosphorescent polymer yellow-light-emitting diodes based on solution-processed small molecular electron transporting layer. ACS Appl. Mater. Interfaces 3, 410–416 (2011). 21299189 10.1021/am1010018

[b42] Lee S., Kim K. H., Limbach D., Park Y. S. & Kim J. J. Low roll-off and high efficiency orange organic light emitting diodes with controlled co-doping of green and red phosphorescent dopants in an exciplex forming co-host. Adv. Funct. Mater. 33, 4105–4110 (2013).

[b43] Glebeler C., Antoniadis H., Bradley D. D. C. & Shirota Y. Space-charge-limited charge injection from indium tin oxide into a starburst amine and its implications for organic light-emitting diodes. Appl. Phys. Lett. 72, 2448–2450 (1998).

[b44] Goushi K., Yoshida K., Sato K. & Adachi C. Organic light-emitting diodes employing efficient reverse intersystem crossing for triplet-to-singlet state conversion. Nat. Photonics 6, 253–258 (2012).

[b45] Seino Y., Sasabe H., Pu Y. J. & Kido J. High-performance blue phosphorescent OLEDs using energy transfer from exciplex. Adv. Mater. 26, 1612–1616 (2014). 24452829 10.1002/adma.201304253

[b46] Zhou D. Y., Siboni H. Z., Wang Q., Liao L. S. & Aziz H. Host to guest energy transfer mechanism in phosphorescent and fluorescent organic light-emitting devices utilizing exciplex-forming hosts. J. Phys. Chem. C 118, 24006–24012 (2014).

[b47] Graves D., Jankus V., Dias F. B. & Monkman A. Photophysical investigation of the thermally activated delayed emission from films of m-MTDATA:PBD exciplex. Adv. Funct. Mater. 24, 2343–2351 (2014).

[b48] Morteani A. C., Sreearunothai P., Herz L. M., Friend R. H. & Silva C. Exciton regeneration at polymeric semiconductor heterojunctions. Phys. Rev. Lett. 92, 247402 (2004). 15245130 10.1103/PhysRevLett.92.247402

[b49] Morteani A. C., Friend R. H. & Silva C. Exciton trapping at heterojunctions in polymer blends. J. Chem. Phys. 122, 244906 (2005). 16035815 10.1063/1.1924504

[b50] Park Y. S., Kim K. H. & Kim J. J. Efficient triplet harvesting by fluorescent molecules through exciplexes for high efficiency organic light-emitting diodes. Appl. Phys. Lett. 102, 153306 (2013).

[b51] Jou J. H. *et al.* Highly efficient yellow organic light emitting diode with a novel wet- and dry-process feasible iridium complex emitter. Adv. Funct. Mater. 24, 555–562 (2014).

[b52] Fan C. & Yang C. L. Yellow/orange emissive heavy-metal complexes as phosphors in monochromatic and white organic light-emitting devices. Chem. Soc. Rev. 43, 6439–6469 (2014). 24927103 10.1039/c4cs00110a

[b53] Kim J. S., Friend R. H., Grizzi I. & Burroughes J. H. Spin-cast thin semiconducting polymer interlayer for improving device efficiency of polymer light-emitting diodes. Appl. Phys. Lett. 87, 023506 (2005).

[b54] Yan H. *et al.* High-performance hole-transport layers for polymer light-emitting diodes. Implementation of organosiloxane cross-linking chemistry in polymeric electroluminescent devices. J. Am. Chem. Soc. 127, 3172–3183 (2005). 15740157 10.1021/ja044455q

[b55] Greenham N. C. & Bobbert P. A. Two-dimensional electron-hole capture in a disordered hopping system. Phys. Rev. B 68, 245301 (2003).

[b56] Ding J. Q. *et al.* Highly efficient green-emitting phosphorescent iridium dendrimers based on carbazole dendrons. Adv. Funct. Mater. 16, 575–581 (2006).

[b57] Ding J. Q. *et al.* Highly efficient phosphorescent bis-cyclometalated iridium complexes based on quinoline ligands. Synth. Met. 155, 539–548 (2005).

[b58] Zhang B. H. *et al.* High-efficiency single emissive layer white organic light-emitting diodes based on solution-processed dendritic host and new orange-emitting iridium complex. Adv. Mater. 24, 1873–1877 (2012). 22410940 10.1002/adma.201104758

[b59] D’Andrade B. W. *et al.* Relationship between the ionization and oxidation potentials of molecular organic semiconductors. Org. Electron. 6, 11–20 (2005).

[b60] Steyrleuthner R., Bange S. & Neher D. Reliable electron-only devices and electron transport in n-type polymers. J. Appl. Phys. 105, 064509 (2009).

