# Supplementary material for: Solution-Processed Phosphorescent Organic Light-Emitting Diodes with Ultralow Driving Voltage and Very High Power Efficiency
Source: Sci Rep. 2015 Jul 24;5:12487. doi: 10.1038/srep12487 (PMC4513343; doi:10.1038/srep12487)
Supplement: Supplementary Information [file srep12487-s1.pdf]

Supplementary information for

Solution-Processed Phosphorescent Organic Light-Emitting Diodes with Ultralow Driving Voltage and Very High Power Efficiency

Shumeng Wang[1,2], Xingdong Wang[1], Bing Yao[1], Baohua Zhang*[1], Junqiao Ding[1], Zhiyuan Xie[1] and Lixiang Wang*[1]

[1]State Key Laboratory of Polymer Physics and Chemistry, Changchun Institute of Applied Chemistry, Chinese Academy of Sciences, Changchun 130022, P. R. China

[2]University of Chinese Academy of Sciences , Beijing 100049, P. R. China

E-mail: bhzhang512@ciac.ac.cn(B. Z); lixiang@ciac.ac.cn(L. W)

Table S1. Comparison of device performance with yellow/orange emissions.

Device type	Voltage (V) 1/100/1000/10000 cd m^{-2}	LE (cd A^{-1}) Max/100/1000/10000 cd m^{-2}	PE (lm W^{-1}) Max/100/1000/10000 cd m^{-2}	EQE (%) Max/100/1000/10000 cd m^{-2}	CIE (x, y)	Ref.
s-PhOLED	2.36/2.60/3.03/4.08	74.3/72.6/70.1/53.5	97.2/87.7/72.5/40.8	25.2/24.8/23.7/18.1	(0.52, 0.47)	This work
s-PhOLED	3.0/4.7/6.7/10.9	49.7/--/40.9/--	43.9/--/19.7/--	17.6/--/14.4/--	(0.53, 0.47)	Ref. S1
s-PhOLED	--/3.2/3.9/5.2	--/65.7/64.7/42.9	--/64.4/52.3/26.1	--/18.9/18.5/12.2	(0.43, 0.56)	Ref. S2
s-PhOLED	--/4.3/5.3/7.0	--/47.6/45.0/34.4	--/35.1/26.7/15.4	--/14.6/13.8/10.5	(0.50, 0.50)	Ref. S3
s-PhOLED	--/--/--/--	41.7/--/--/--	12.5/--/--/--	12.7/--/--/--	--	Ref. S4
s-PhOLED	3.9/--/--/--	40.4/--/38.8/--	15.8/--/11.9/--	12.3/--/11.8/--	(0.51, 0.48)	Ref. S5
v-PhOLED	--/2.8/3.3/5.3	--/75.9/77.9/61.0	--/84.8/75.1/36.6	--/22.1/22.6/17.6	(0.45, 0.54)	Ref. S2
v-PhOLED	--/3.1/3.8/5.6	--/77/71/56	--/79/59/31	--/25/23/18	--	Ref. S6
v-PhOLED	2.4/--/--/--	--/--/--/--	62.1--/49.9/--	25.0/--/24.3/21.2	--	Ref. S7
v-PhOLED	4/5.1/5.9/7.7	71.6/--/--/--	44.5/--/--/--	24.9/--/--/--	(0.50, 0.49)	Ref. S8
v-PhOLED	2.9/3.8/5.0/8.1	52.4/52.3/50.4/--	48.7/44.4/31.0/--	17.9/17.9/17.2/--	(0.51, 0.49)	Ref. S9
v-PhOLED	4.2/6.0/7.2/9.4	87.9/--/--/--	46.0/--/--/--	29.6/--/--/--	(0.49, 0.50)	Ref. S10

s-PhOLED: solution-processed electrophosphorescent devices; v-PhOLED: vacuumn-deposited electrophosphorescent devices.

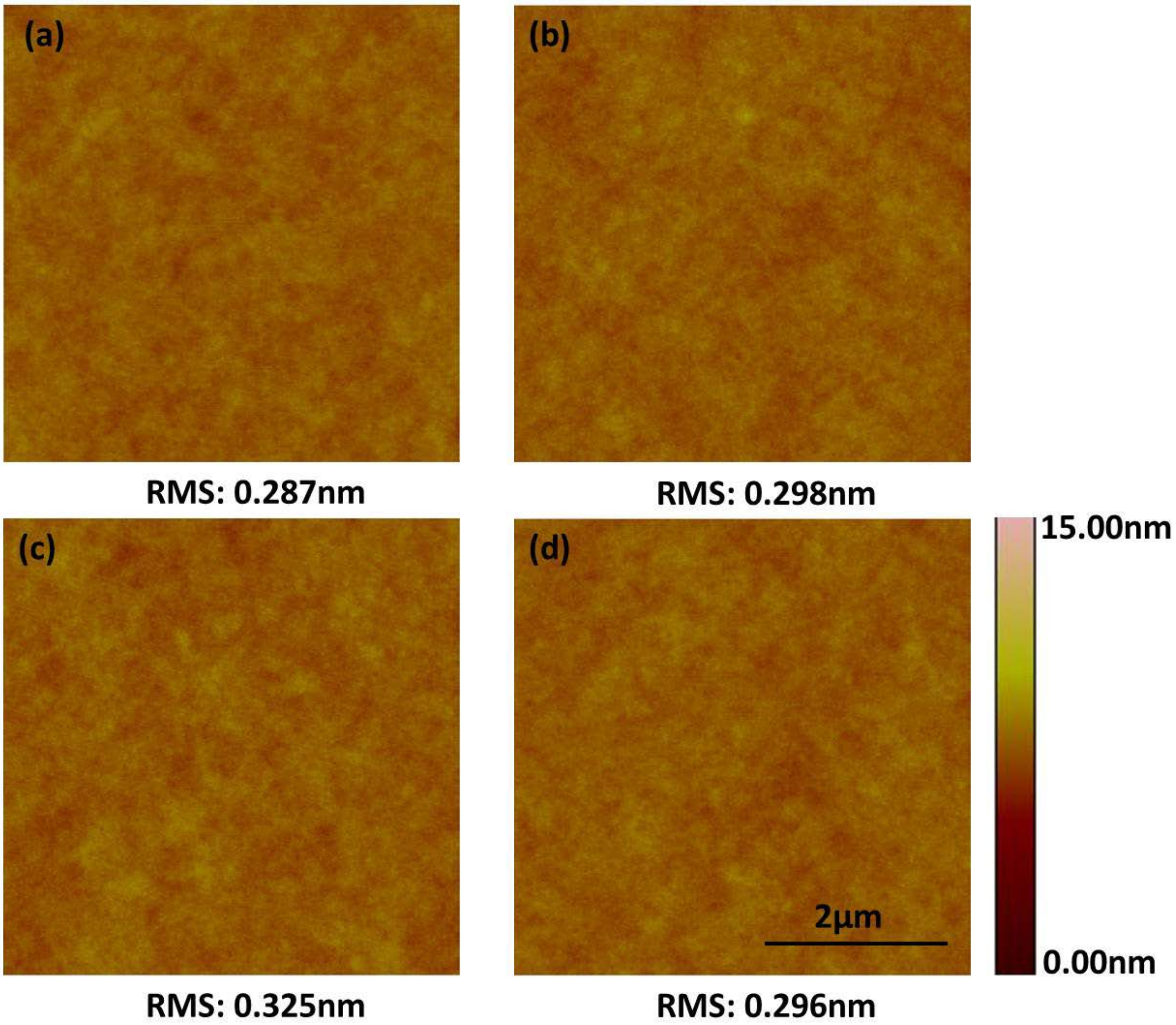

Figure S1. AFM morphologies of pristine m-MTDATA(a), m-MTDATA:Ir(Flpy-CF_3)$_3$(1 wt.%)(b), m-MTDATA:G0(10 wt.%)(c), and m-MTDATA:Ir(TPAPQ)$_2$acac(5 wt.%)(d) films. As shown, all m-MTDATA films (with or without doping phosphor emitters) show comparable and well smooth morphologies with extremely low RMS roughness. It indicates the excellent film-forming capability of m-MTDATA for solution-processed phosphorescent OLEDs.

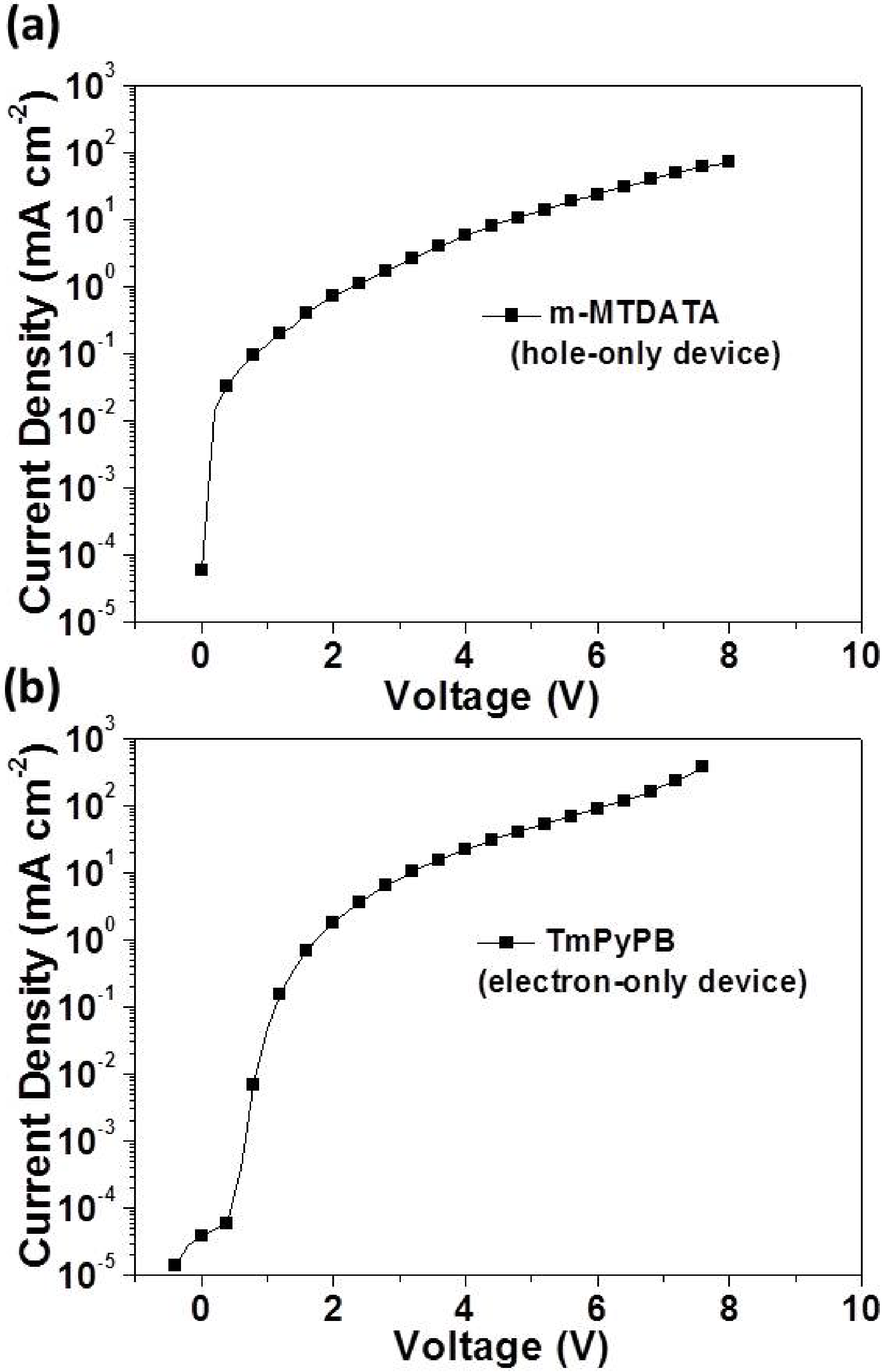

Figure S2. Current density-voltage characteristics for hole-only device of pure m-MTDATA (a), and electron-only device of pure TmPyPB (b).

Table S2. Summery of cyclic voltammetry (CV) and photophysical properties for m-MTDATA ,TmPyPB, G0, Ir(Flpy-CF_3)$_3$, and Ir(TPAPQ)$_2$acac.

Material	HOMO [a] (eV)	LUMO [a] (eV)	HOMO [c] (eV)	LUMO [c] (eV)	S_1 [d] (eV)	T_1 [e] (eV)	Φ_p [f]
m-MTDATA	-4.68	-1.50 [b]	-5.10	-1.92	2.89	2.66	--
TmPyPB	-6.21	-2.12	-6.63	-2.54	3.47	2.80	--
G0	-4.84	-2.16	-5.26	-2.58	--	2.45	0.41
Ir(Flpy-CF_3)$_3$	-5.16	-2.72	-5.58	-3.14	--	2.24	0.52
Ir(TPAPQ)$_2$acac	-5.07	-2.86	-5.49	-3.28	--	2.07	0.25

[a] The HOMO and LUMO energy levels are measured by the CV and calculated according to the equations: E_{HOMO} = -e [4.8V +E_{ox}] and E_{LUMO} = -e [4.8V +E_{red}], respectively; [b] The LUMO energy level of m-MTDATA is calculated according to the equation: E_{LUMO}= E_{HOMO} +E_g; [c] With the widely used HOMO energy level (-5.10 eV) of m-MTDATA as the reference that is measured by ultraviolet photoemission spectroscopy (UPS), the HOMO and LUMO energy levels are adjusted according to the linear relationship between the CV and UPS results; [d] Energy of a fluorescence peak wavelength positioned on the shortest wavelength side for films; [e] Energy of a phosphorescence peak wavelength positioned on the shortest wavelength side for toluene solution (4x10^{-4} M) at 77K; [f] Solution PL quantum efficiency was measured in argon atmosphere by a relative method using fac-Ir(ppy)$_3$ (Φ_p = 0.40 in toluene) as the standard.

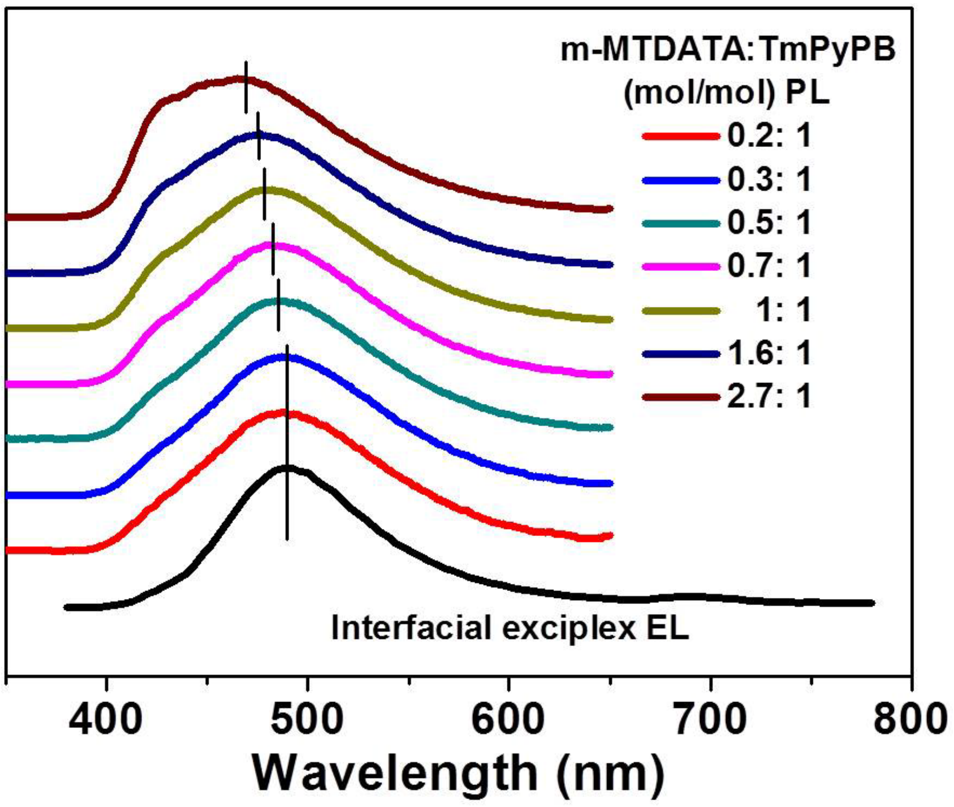

Figure S3. Normalized PL spectra of m-MTDATA:TmPyPB blended films with different ratios as well as normalized EL spectrum of m-MTDATA/TmPyPB bilayer device.

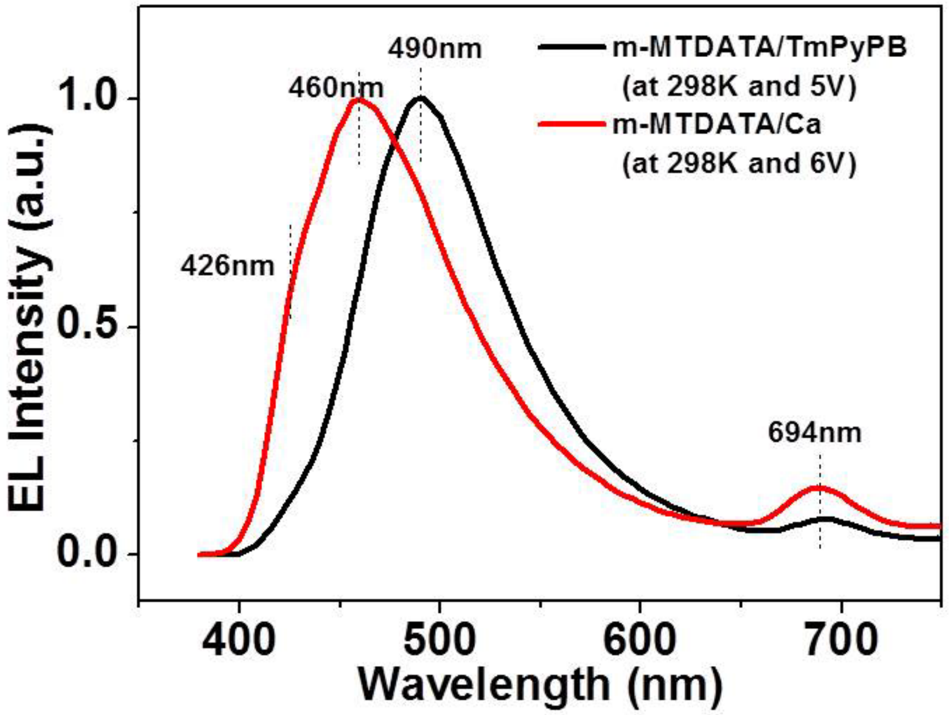

Figure S4. EL spectra of single layer device (ITO/PEDOT:PSS(40 nm)/m-MTDATA(70 nm)/Ca(5 nm)/Al(100 nm)) and bilayer device A (0 wt.% Ir(Flpy-CF_3)$_3$) (ITO/PEDOT:PSS(40 nm)/m-MTDATA(40 nm)/TmPyPB(55 nm)/LiF(0.5 nm)/Al(100 nm)). The emssion peak at 426nm and 460nm is ascribed to m-MTDATA exciton emission, although the relative intensity of the two peaks changes compared with the PL spectrum of pure m-MTDATA film. Emission peak at 694nm appears both in the single layer device and bilayer device, and does not exist in the PL spectra of pure m-MTDATA and m-MTDATA:TmPyPB(1:1, mol/mol) films. So it could be ascribed to m-MTDATA electromer emission.

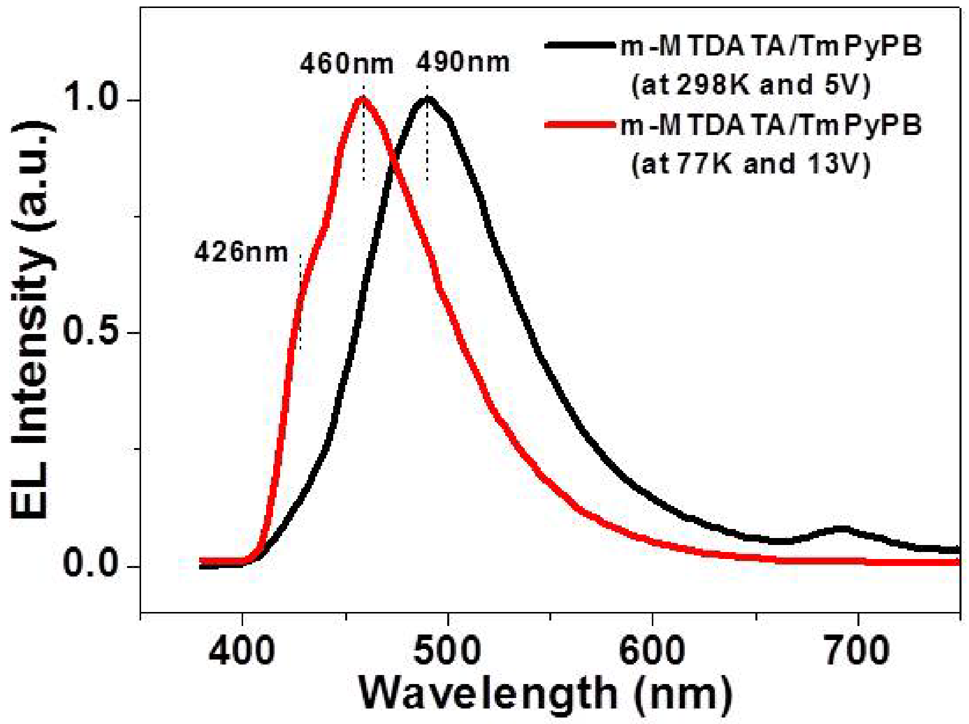

Figure S5. EL spectra of bilayer device A (0 wt.% Ir(Flpy-CF_3)$_3$) (ITO/PEDOT:PSS(40 nm)/m-MTDATA(40 nm)/TmPyPB(55 nm)/LiF(0.5 nm)/Al(100 nm)) at 5V(298K) and 13V(77K). The main peak of EL spectrum at 5V is the emission of interfacial exciplex. The EL spectrum at 13V is almost the same to m-MTDATA single layer device (ITO/PEDOT:PSS(40 nm)/m-MTDATA(70 nm)/Ca(5 nm)/Al(100 nm)) suggesting that electron injects into m-MTDATA and bulk exciton forms at high electric field. But for device A (1 wt.% Ir(Flpy-CF_3)$_3$), it achieves a luminance of 20000cd m^{-2} below 5V. So in the operating condition, hole and electron capturing across the interface to form exciplex is the exciton formation way without m-MTDATA bulk exciton existense.

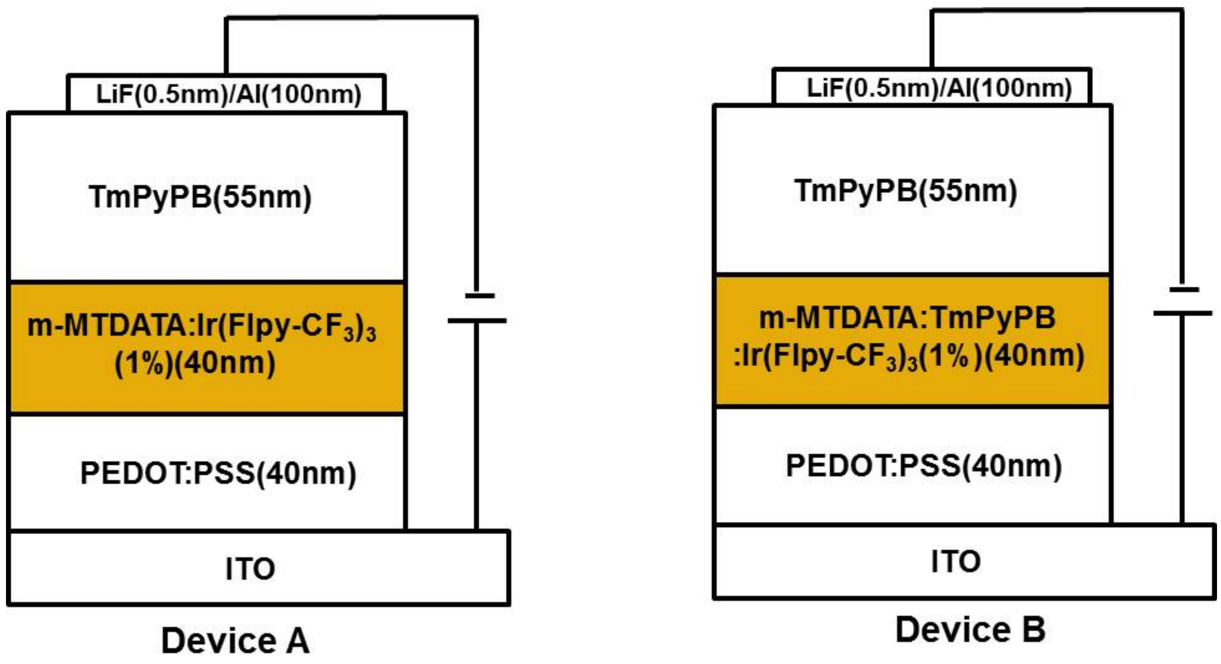

Figure S6. Device structures of device A (1 wt.% Ir(Flpy-CF_3)$_3$) and B (1 wt.% Ir(Flpy-CF_3)$_3$) (Device A: ITO/PEDOT:PSS(40 nm)/m-MTDATA:Ir(Flpy-CF_3)$_3$(1 wt.%)(40 nm)/TmPyPB(55 nm)/LiF(0.5 nm)/Al(100 nm) and Device B: ITO/PEDOT:PSS(40 nm)/m-MTDATA:TmPyPB (1:1, mol/mol):Ir(Flpy-CF_3)$_3$ (1 wt.%)(40 nm)/TmPyPB(55 nm)/LiF(0.5 nm)/Al(100 nm)).

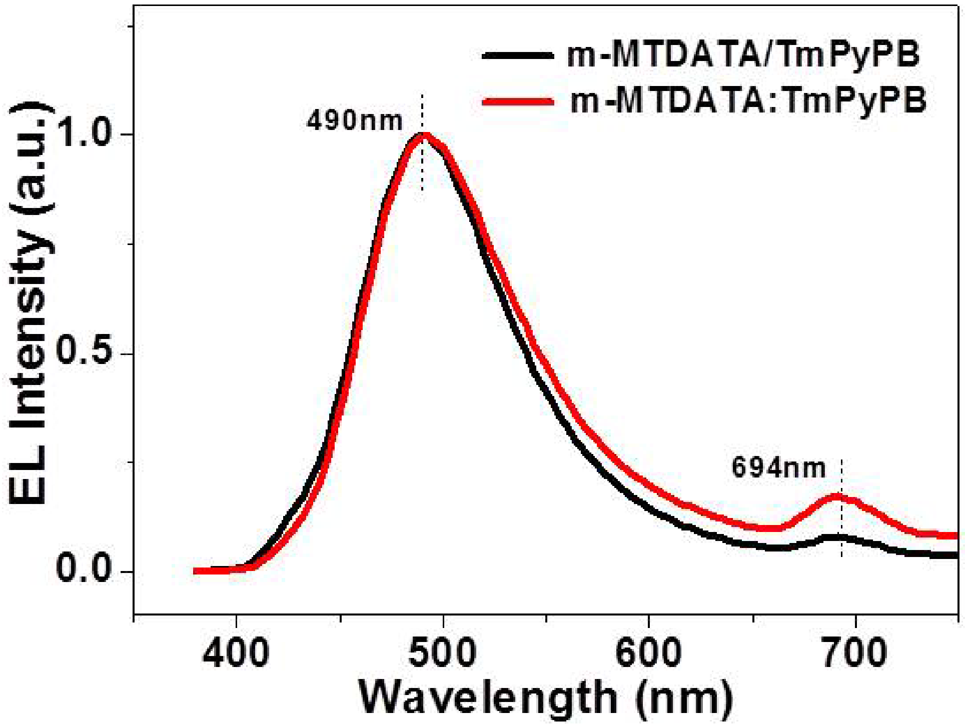

Figure S7. EL spectra of interfacial exciplex device A (0 wt.% Ir(Flpy-CF_3)$_3$) (ITO/PEDOT:PSS(40 nm)/m-MTDATA(40 nm)/TmPyPB(55 nm)/LiF(0.5 nm)/Al(100 nm)) and bulk exciplex device B (0 wt.% Ir(Flpy-CF_3)$_3$) (ITO/PEDOT:PSS(40 nm)/m-MTDATA:TmPyPB(1:1, mol/mol)(40 nm)/TmPyPB(55 nm)/LiF(0.5 nm)/Al(100 nm)). The main peak of EL spectra for bulk device B (0 wt.% Ir(Flpy-CF_3)$_3$) is also the emission of exciplex and almost the same to EL spectra of interfacial device A (0 wt.% Ir(Flpy-CF_3)$_3$).

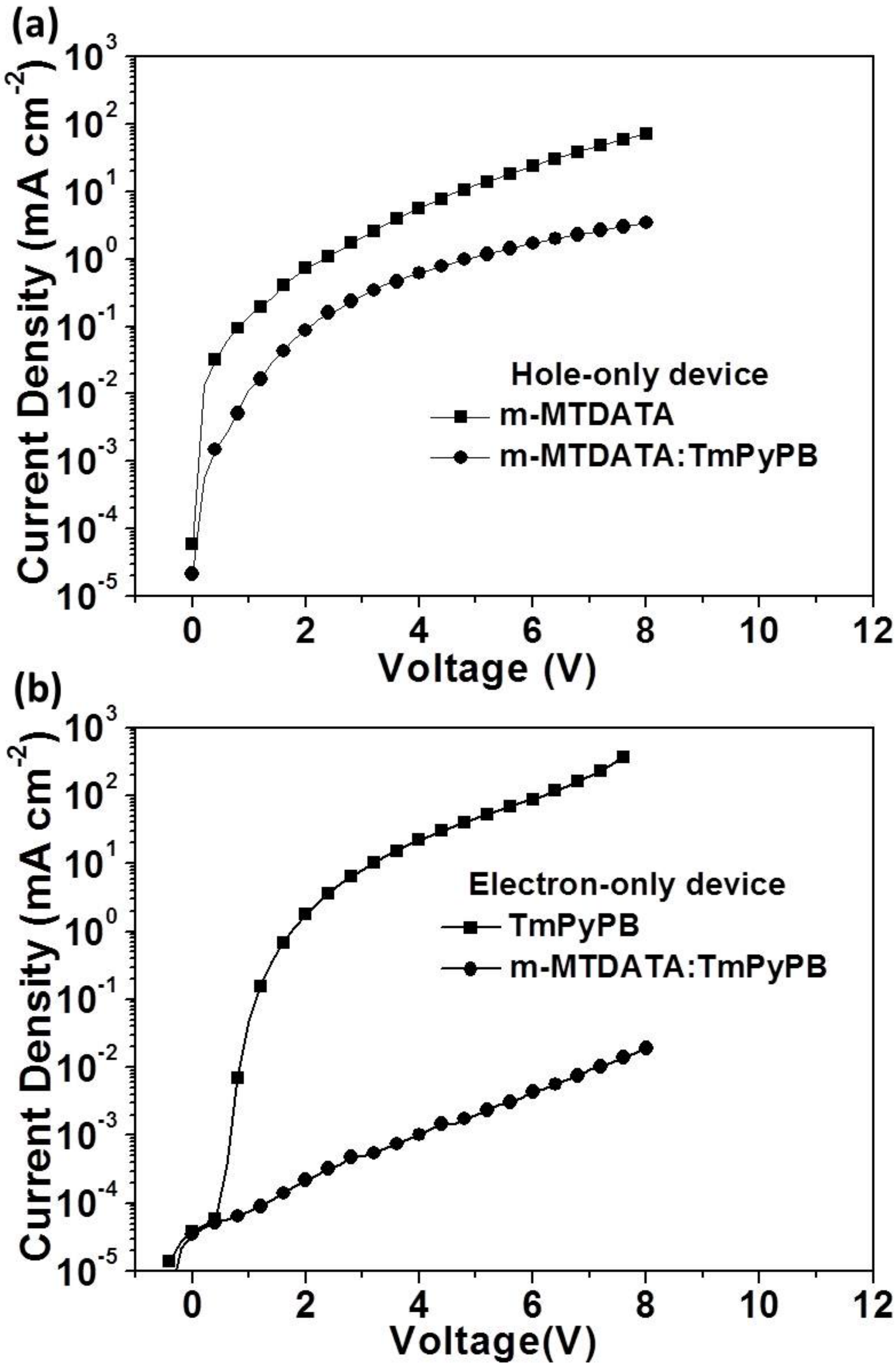

Figure S8. (a) Current density-voltage characteristics for hole-only devices of pure m-MTDATA and m-MTDATA:TmPyPB(1:1, mol/mol) films; (b) Current density-voltage characteristics for electron-only devices of pure TmPyPB and m-MTDATA:TmPyPB(1:1, mol/mol) films.

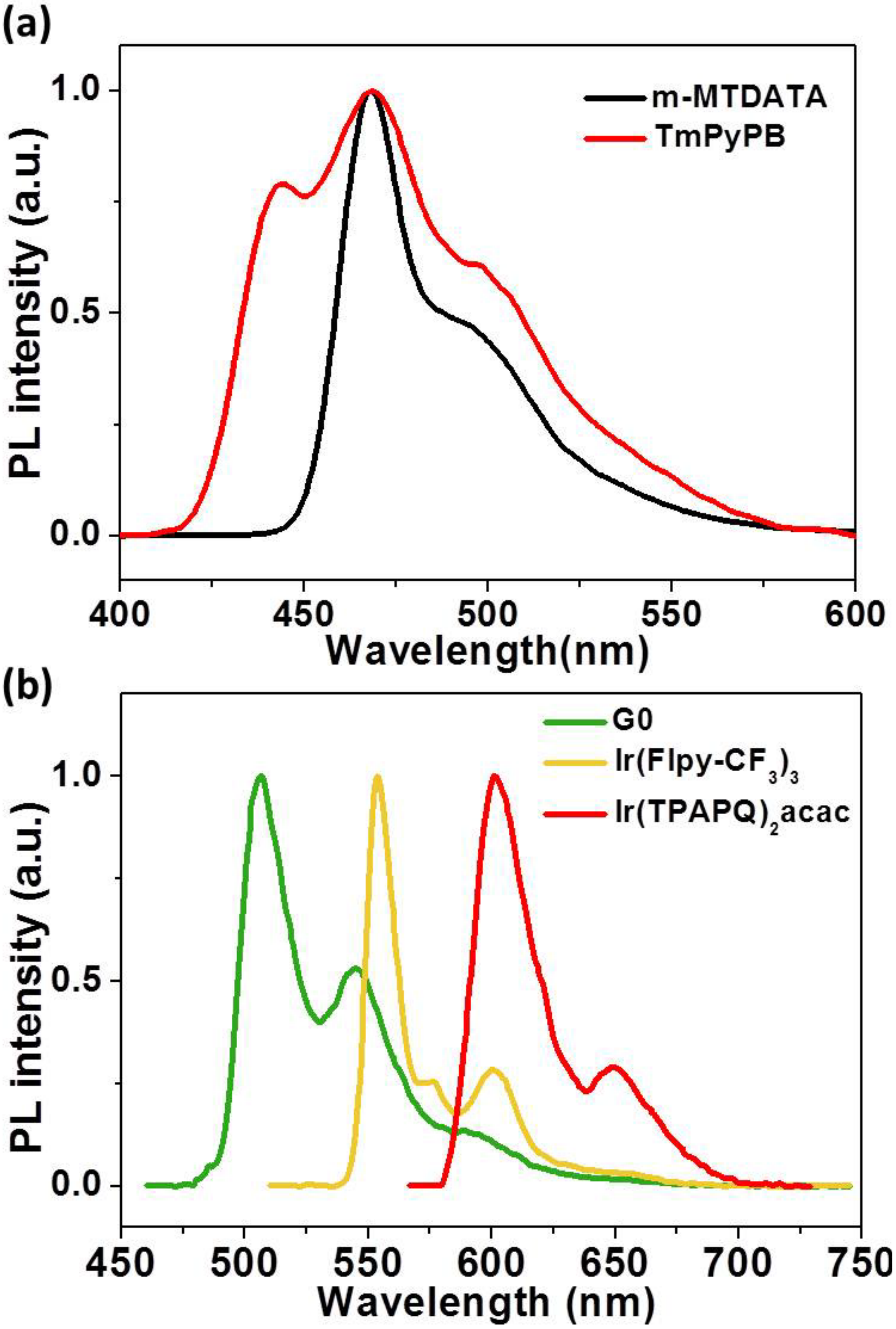

Figure S9. (a) Phosphorescence spectra of m-MTDATA and TmPyPB in toluene solution ($4x10^{-4}$M) at 77K; (b) Phosphorescence spectra of G0, Ir(Flpy-CF_3)$_3$, and Ir(TPAPQ)$_2$acac in toluene solution ($4x10^{-4}$M) at 77K.

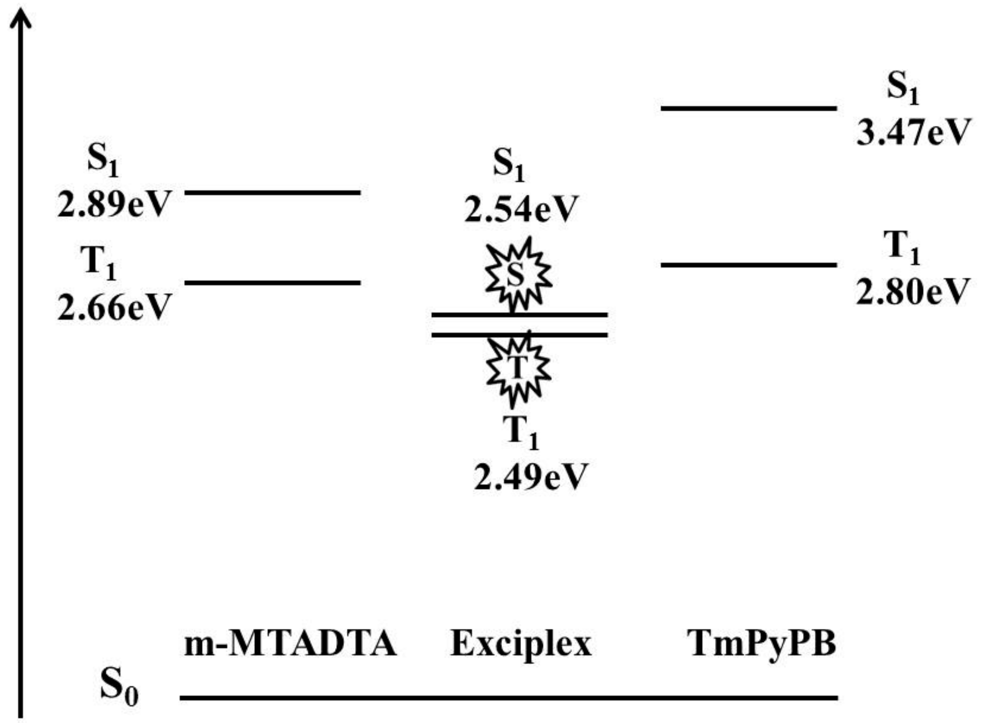

Figure S10. Schematic diagram of energy levels for m-MTDATA, TmPyPB, and exciplex (Triplet energy level is calculated assuming $\triangle E_{st}$=50meV for almost zero exchange energy exciplex materials).

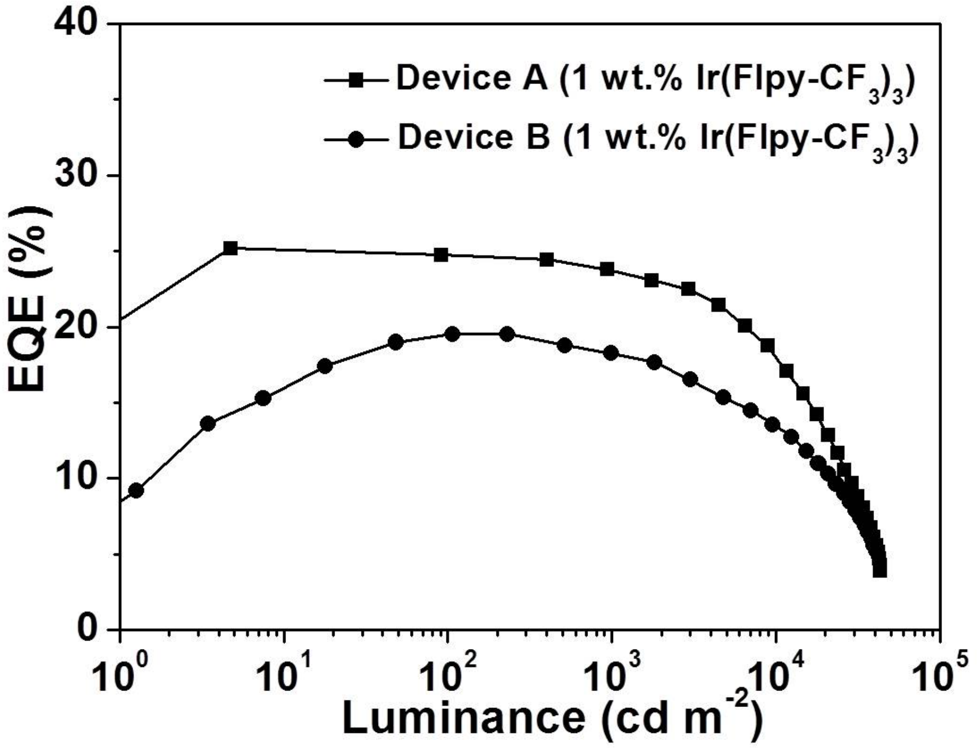

Figure S11. EQE-luminance characteristics for device A (1 wt.% Ir(Flpy-CF_3)$_3$) and device B (1 wt.% Ir(Flpy-CF_3)$_3$).

As shown, the roll off (up to ca. 1000 cd m^{-2}) is actually low, which is ascribed to the very limited polaron-exciton quenching in view of the property of interfacial exciplex. As for the relative high roll-off rate at even higher luminance(e.g.10000 cd m^{-2}), the most possible reason should be attributed to triplet-triplet annihilation in the vicinity of m-MTDATA:dopant/TmPyPB interface, which is caused by the accumulation of triplet excitons at high current density. And the roll off is not due to transition in energy transfer from exciplex-dopant to bulk exciton formation since bulk exciton emission actually do not be observed even at 5V(corresponding to high luminance of ca.24000 cd m^{-2}).

(a)

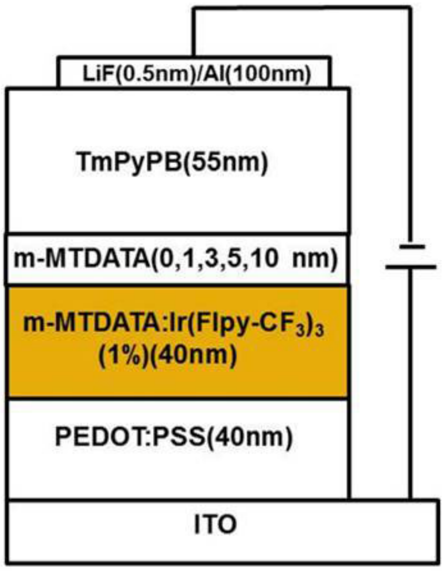

(b)

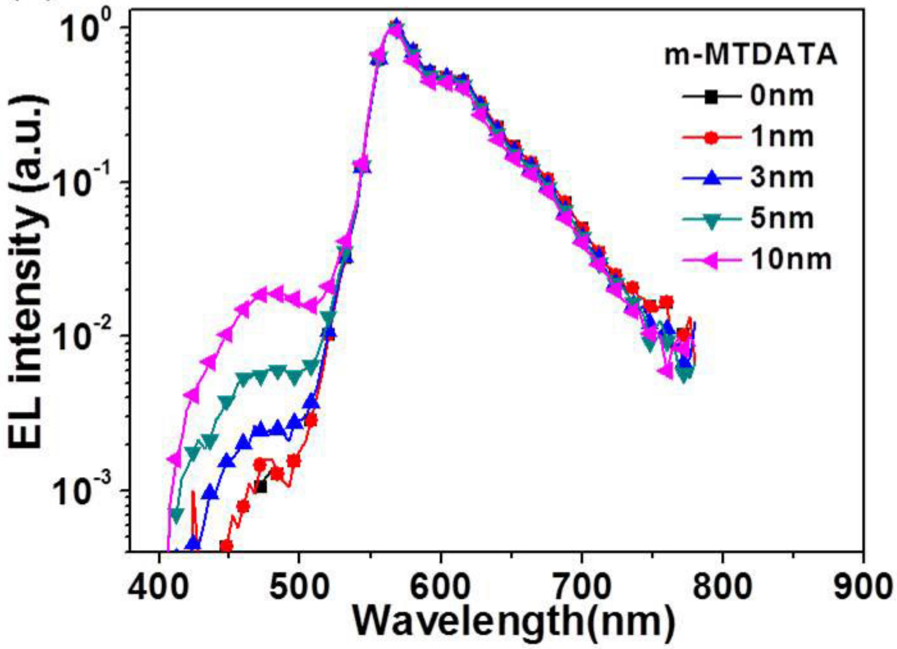

Figure S12. Device structures of of s-PhOLEDs with non-doped m-MTDATA layer (a), and their corresponding EL spectra at 5V in a semi-log plot compared with device A (b).

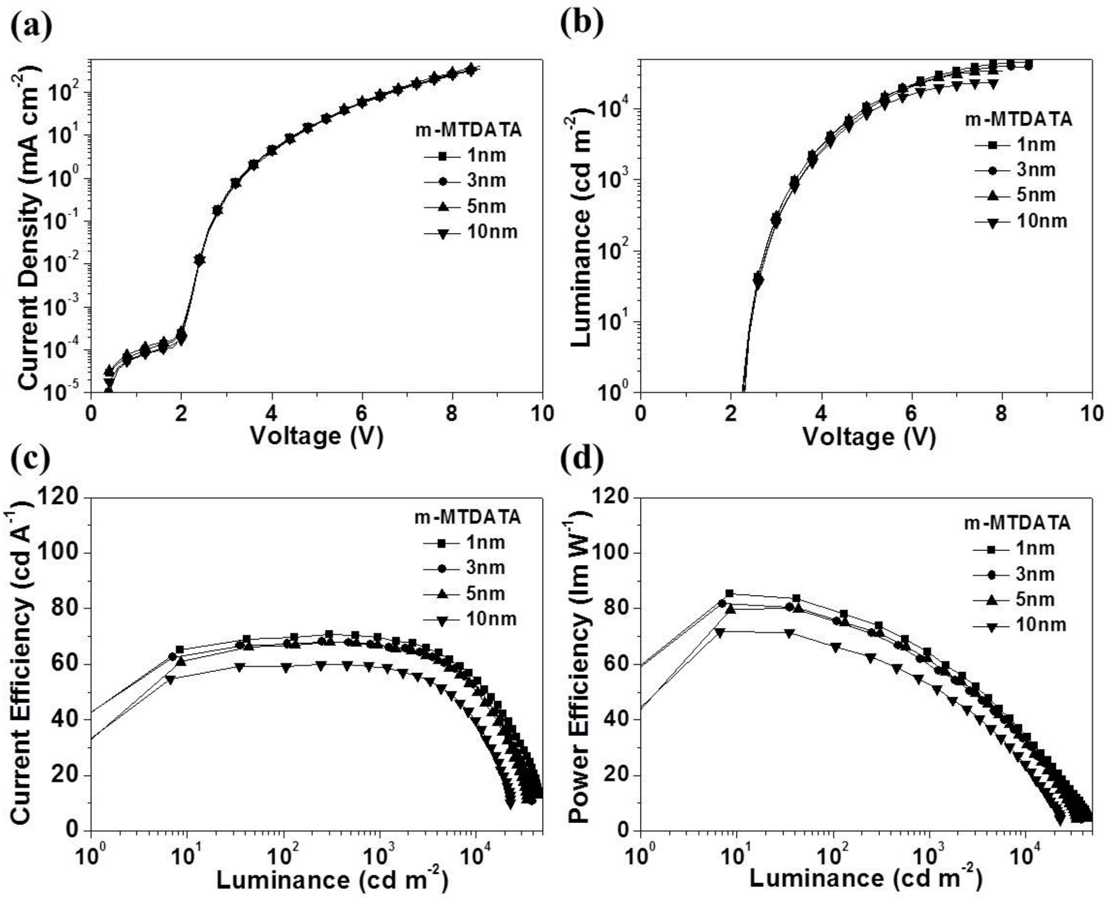

Figure S13. Current density-voltage characteristics (a), luminance-voltage characteristics (b), luminous efficiency-luminance characteristics (c) and power efficiency-luminance characteristics (d) for devices with non-doped m-MTDATA layer.

Table S3. Device performance of s-PhOLEDs with non-doped m-MTDATA layer.

Device	Voltage (V)	LE (cd A^{-1})	PE (lm W^{-1})	EQE (%)
	Turn-on/100/1000 cd m^{-2}	100/ 1000cd m^{-2}	100/ 1000cd m^{-2}	100/1000 cd m^{-2}
0 nm	2.36/2.60/3.03	72.6/70.1	87.7/72.5	24.8/23.7
1 nm	2.34/2.76/3.42	69.5/69.6	79.4/64.2	23.6/23.7
3 nm	2.342.76/3.42	67.4/66.9	76.2/61.4	22.8/22.6
5 nm	2.34/2.80/3.51	67.3/66.9	76.2/59.8	22.8/22.6
10 nm	2.34/2.80/3.51	59.0/58.8	66.6/52.6	19.2/19.1

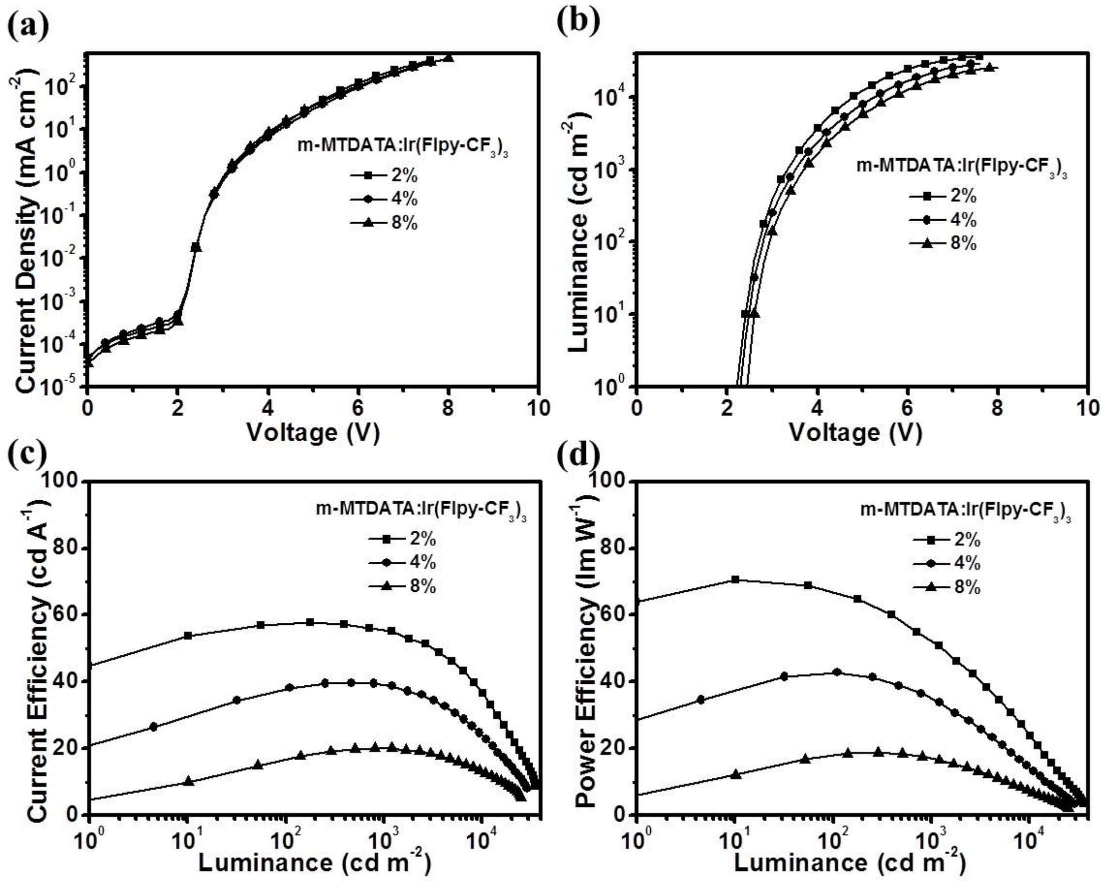

Figure S14. Current density-voltage characteristics (a), luminance-voltage characteristics (b), luminous efficiency-luminance characteristics (c) and power efficiency-luminance characteristics (d) for device A with different doping concentration above 1wt.%. As increasing the doping concentrations, J-V curves remain nearly unchanged, indicating the trap-free behaviors for device A, just as we disscussed in the main text. But the L-V curves decrease gradually, leading to the reduction of current efficiency and power efficiency.

Table S4. Device performance of device A with different doping concentrations.

Device	Voltage (V)	LE (cd A^{-1})	PE (lm W^{-1})	EQE (%)
	Turn-on/100/1000 cd m^{-2}	100/ 1000cd m^{-2}	100/ 1000cd m^{-2}	100/1000 cd m^{-2}
1%	2.36/2.60/3.03	72.6/70.1	87.7/72.5	24.8/23.7
2%	2.32/2.70/3.28	57.3/55.6	66.8/52.9	19.4/18.7
4%	2.33/2.75/3.50	37.5/39.1	42.7/35.2	12.4/13.2
8%	2.35/2.92/3.72	16.3/20.1	17.6/17.2	5.5/6.8

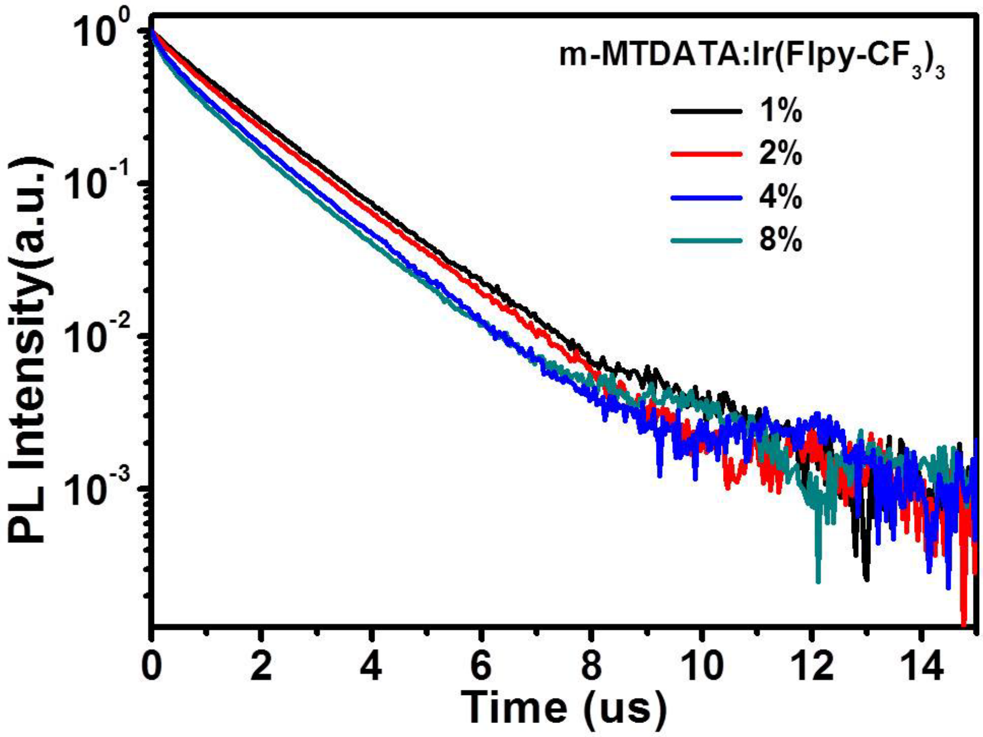

Figure S15. Transient photoluminescent results for m-MTDATA:Ir(Flpy-CF_3)$_3$ films with different doping concentrations (1,2,4,8 wt. %). All thes films were excited at 460nm. At this condition, only Ir(Flpy-CF_3)$_3$ was motivated because there is no absorption for m-MTDATA host as this wavelength. After the motivation, two main process may occur for the generated Ir(Flpy-CF_3)$_3$ excitons. One is the intrinsic radiative emission after exciation and the other is annihilation induced by self-aggregation of Ir(Flpy-CF_3)$_3$. As the doping concentration increasing from 1 wt% to 8 wt.%, the exciton lifetimes of Ir(Flpy-CF_3)$_3$ was monotonically decreased, indicating that aggregation induced exciton annihilation process becomes more and more serious. Such results are well consistent with the device results shown in Figure S14 and Table S4.

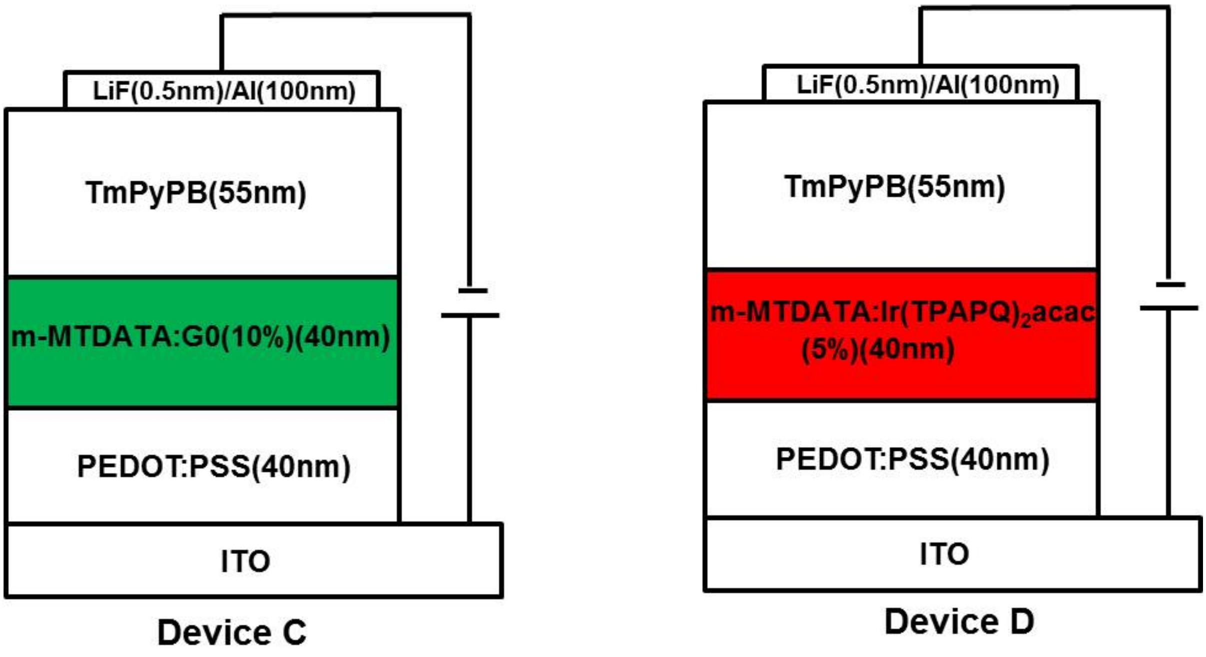

Figure S16. Device structures of device C and D with G0 and Ir(TPAPQ)$_2$acac dopants (Device C: ITO/PEDOT:PSS(40 nm)/m-MTDATA:G0(10 wt.%)(40 nm)/TmPyPB(55 nm)/LiF(0.5 nm)/Al(100 nm) and Device D: ITO/PEDOT:PSS(40 nm)/m-MTDATA:Ir(TPAPQ)$_2$acac(5 wt.%)(40 nm)/TmPyPB(55 nm)/LiF(0.5 nm)/Al(100 nm)).

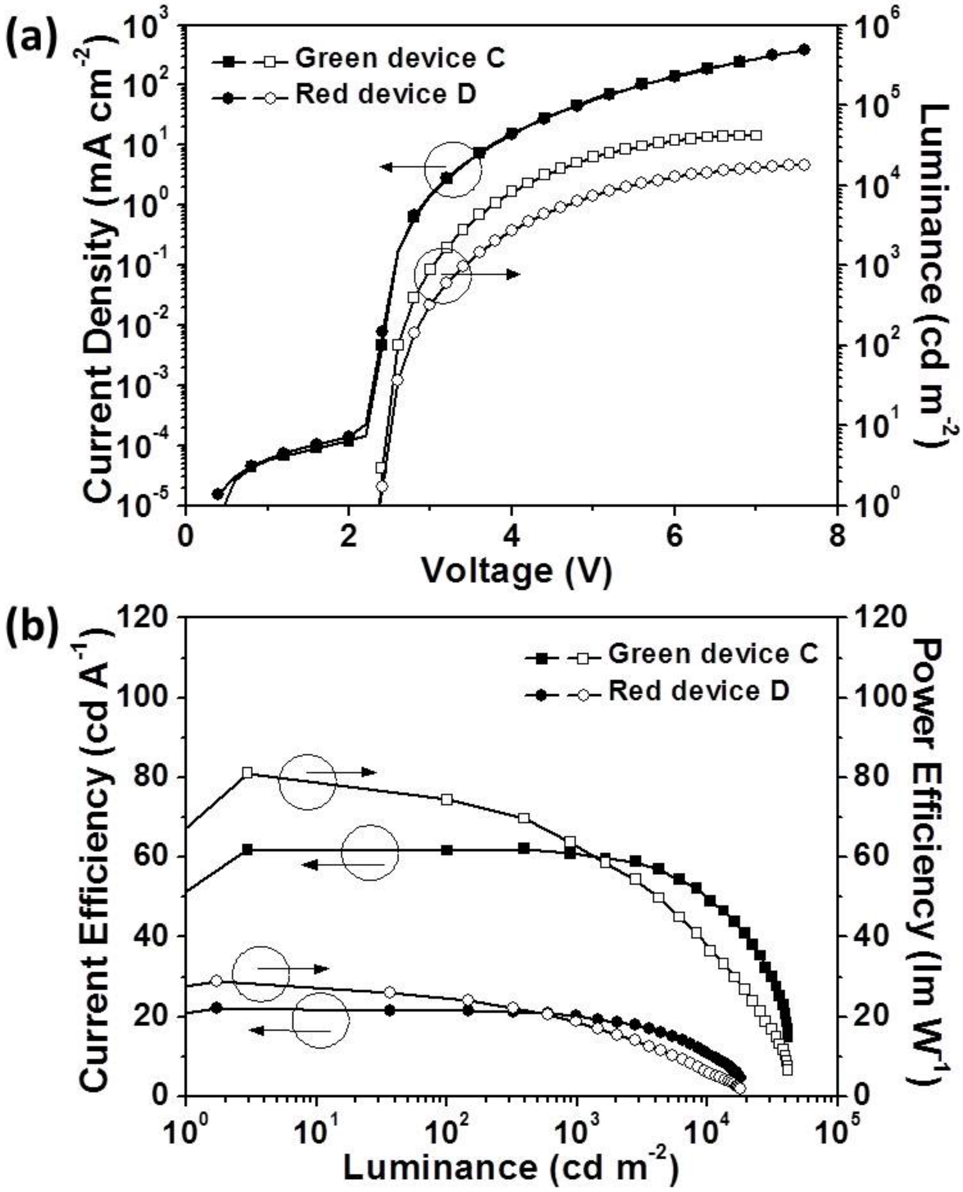

Figure S17. (a) Current density-voltage-luminance characteristics for device C and device D; (b) Current efficiency-luminance-power efficiency characteristics for device C and device D.

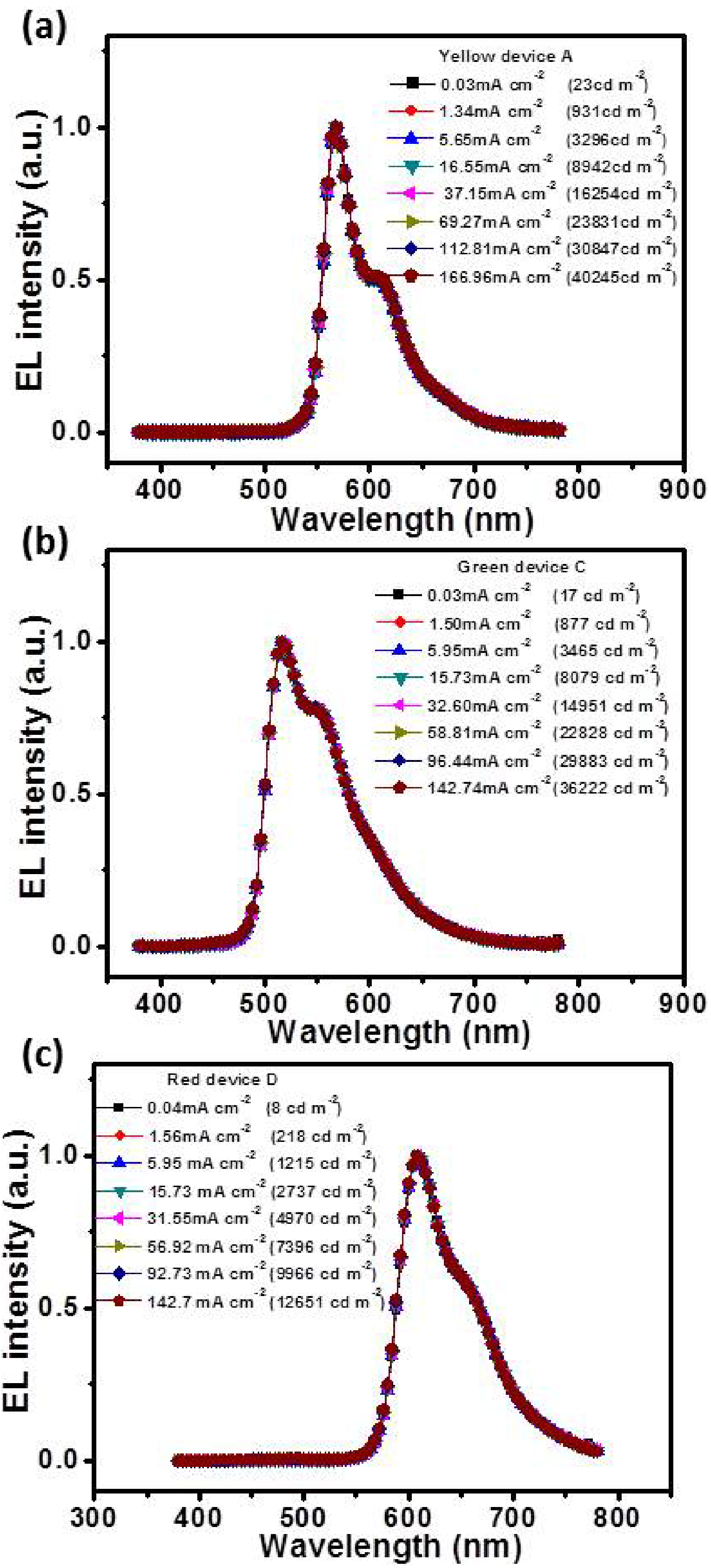

Figure S18. EL spectra of yellow device A, green device C, and red device D at different current density and luminance.

References:

S1. Zhang, B. H. *et al*. High-efficiency single emissive layer white organic light-emitting diodes based on solution-processed dendritic host and new orange-emitting iridium complex. *Adv. Mater.* **24,** 1873-1877 (2012).

S2. Jou, J. H. *et al*. Highly efficient yellow organic light emitting diode with a novel wet- and dry-process feasible iridium complex emitter. *Adv. Funct. Mater.* **24,** 555-562 (2014).

S3. Jou, J. H., *et al*. Using light-emitting dyes as a co-host to markedly improve efficiency roll-off in phosphorescent yellow organic light emitting diodes. *J. Mater. Chem. C* **1,** 394-400 (2013).

S4. Ye, T., Shao, S., Chen, J., Wang, L., & Ma, D. Efficient phosphorescent polymer yellow-light-emitting diodes based on solution-processed small molecular electron transporting layer. *ACS Appl. Mater. Interfaces* **3,** 410-416 (2011).

S5. Wu, H. B. *et al*. Efficient polymer white-light-emitting devices for solid-state lighting, *Adv. Mater.* **21,** 4181-4184 (2009).

S6. Jou J. H. *et al*. High efficiency yellow organic light emitting diodes with a balanced carrier injection co-host structure. *J. Mater. Chem. C* **1,** 5110-5115 (2013).

S7. Lee, S., Kim, K. H., Limbach, D., Park, Y. S., & Kim, J. J. Low roll-off and high efficiency orange organic light emitting diodes with controlled co-doping of green and red phosphorescent dopants in an exciplex forming co-host. *Adv. Funct. Mater.* **33,** 4105-4110 (2013).

S8. Wang, R. J. *et al*. Highly efficient orange and white organic light-emitting diodes based on new orange iridium complexes. *Adv. Mater.* **23,** 823-2827 (2011).

S9. Gong, S. L. *et al*. Bipolar tetraarylsilanes as universal hosts for blue, green, orange, and white electrophosphorescence with high efficiency and low efficiency roll-Off. *Adv. Funct. Mater.* **21,** 1168-1178 (2011).

S10. Wang, R. J. *et al*. Homoleptic tris-cyclometalated iridium complexes with 2-phenylbenzothiazole ligands for highly efficient orange OLEDs. *J. Mater. Chem.* **21,** l15494-15500 (2011).
